# Degradation pathway of (R)-1,3-butanediol in *Pseudomonas putida* KT2440 and development of its biosensor

**DOI:** 10.1128/aem.00900-26

**Published:** 2026-06-22

**Authors:** Nandakumar Arumugam, Tayyab Islam, Joon Young Park, Minchang Jang, Mugesh Sankaranarayanan, Donghyuk Kim, Sung Kuk Lee, Sunghoon Park

**Affiliations:** 1 School of Energy and Chemical Engineering, Ulsan National Institute of Science and Technology 131639, Ulsan, Republic of Korea; 2 R&D Center, ACTIVON Co., Ltd., Cheongju, Republic of Korea; 3 Center for Bio-based Chemistry, Korea Research Institute for Chemical Technology, Ulsan, Republic of Korea; 4 Center for Metabolic Engineering & Synthetic Biology, Department of Biotechnology, Vel Tech Rangarajan Dr. Sagunthala R&D Institute of Science and Technology 373270, Chennai, India; Shanghai Jiao Tong University, Shanghai, China

**Keywords:** 1,3-butanediol, microbial degradation, biosensor, GFP fluorescence, mRNA expression

## Abstract

1,3-Butanediol (1,3-BDO) is widely used in consumer and industrial products; however, its microbial degradation remains poorly understood. Here, we dissect the catabolic and regulatory mechanisms of (R)−1,3-BDO utilization in *Pseudomonas putida* KT2440 and develop (R)−1,3-BDO-responsive transcriptional biosensors. Transcriptomics and qRT-PCR revealed strong induction of the *ped* gene cluster, which oxidizes (R)−1,3-BDO to (R)−3-hydroxybutyrate [(R)−3-HB], and of the LysR-regulated operon *PP_2047–2051*, which channels (R)−3-HB toward acetoacetate and acetyl-CoA. Gene deletion and enzyme assays identified *pedE* and *PP_2049* as essential for (R)−1,3-BDO catabolism, with PP_2049 playing a more critical role than the canonical β-hydroxybutyrate dehydrogenase HbdH (*PP_3073*). Regulatory analysis showed that PedR1 directly activates catabolic genes independently of PedR2—challenging the widely accepted indirect-only model—while PedS1 proved dispensable, implying an alternative sensor kinase. Promoter–GFP fusions demonstrated that full activation of the *pedE* promoter requires both PedR1 and the PedS2–PedR2 two-component system, whereas the *pedS2R2* promoter requires only PedR1 and functions in both native and heterologous hosts, including *Escherichia coli*. These results define the genetic and regulatory architecture of (R)−1,3-BDO degradation in *P. putida* and establish *pedE*- and *pedS2R2*-based promoters as (R)−1,3-BDO-responsive biosensors.

**IMPORTANCE:**

1,3-Butanediol is an increasingly prevalent industrial chemical whose environmental fate depends on microbial degradation. Understanding how microorganisms sense and metabolize this compound is therefore essential for both environmental microbiology and biotechnology. This study clarifies the genetic and regulatory basis of (R)-1,3-BDO catabolism in *Pseudomonas putida*, identifying key enzymes and transcriptional regulators that control its utilization. By coupling pathway elucidation with promoter characterization, this work enables the development of (R)-1,3-BDO-responsive biosensors that function in both native and heterologous hosts. These insights are important for understanding the environmental fate of 1,3-BDO and designing inducible regulatory systems for biotechnological applications.

## INTRODUCTION

1,3-Butanediol (1,3-BDO) is a multifunctional chemical widely used in the production of cosmetics, personal care products, pharmaceuticals, and specialty materials, where it serves as a humectant, solvent, and chemical intermediate (1). Due to its widespread use, 1,3-BDO is frequently released into the environment through product disposal and wastewater. It may also be produced as a degradation product during microbial breakdown of certain polyesters and polyurethanes (2). Understanding its microbial degradation is therefore important for evaluating its environmental impact and for advancing biotechnological applications such as bioconversion, biosensing, and dynamic gene regulation. While the catabolic pathways of some diol compounds like ethylene glycol, 1,3-propanediol (1,3-PDO), and 1,4-butanediol (1,4-BDO) have been well-characterized (3–5), microbial degradation of 1,3-BDO remains less studied.

Species of the *Pseudomonas* genus are widely recognized for their roles in the biodegradation of environmental pollutants. In particular, *P. aeruginosa* and *P. putida* KT2440 can oxidize aliphatic alcohols to corresponding acids via a variety of alcohol and aldehyde dehydrogenases (6–8). Prominent among these are PedE and PedH alcohol dehydrogenases, encoded in the so-called *ped* cluster (9). The *ped* gene cluster in *P. putida* and *P. aeruginosa* is regulated by a hierarchical dual control system involving a top transcriptional activator, PedR1 (also known as AgmR), and a down two-component system (TCS) (PedS2/PedR2) (10). PedR1 induces the *pedS2-pedR2* operon (analogous to *exaDE* in *P. aeruginosa*), where PedS2 becomes autophosphorylated under lanthanide-free conditions and activates PedR2, which induces the expression of *pedE*, a calcium-dependent quinoprotein alcohol dehydrogenase (11, 12).

Structurally, 1,3-BDO contains a secondary alcohol group and exists as R- and S-enantiomers. While chemical synthesis typically yields racemic mixtures, highly pure (R)−1,3-BDO can be produced through microbial fermentation. Biotechnological production routes involving the *phaA*-dependent and DERA pathways have been described for this purpose (13, 14). Efficient biosensors for BDO would enable dynamic regulation of production pathways and high-throughput screening of pathway variants (15). Although highly efficient sensors are available for organic acids such as 3-hydroxypropionic acid (3-HP), biosensors for alcohols remain limited. A 1,4-BDO sensor has been reported, but its performance was not fully satisfactory owing to negative regulation and a fold change of less than 10 (16).

In this study, we investigated the degradation pathway of (R)−1,3-BDO and its induction in *P. putida* KT2440. We first confirmed that KT2440 can grow efficiently on (R)−1,3-BDO or (R)−3-HB and then conducted RNA-seq experiments to identify the genes and operons induced during growth on (R)−1,3-BDO and (R)−3-HB. Based on further identification by RT-PCR, gene knockout, and enzyme activity assay, we proposed a corresponding degradation pathway. In addition, we explored the regulatory roles of PedR1 and PedR2 using a GFP-based biosensor system responsive to (R)−1,3-BDO and demonstrated that the induction of the *ped* operons requires regulatory proteins. The *ped* promoters were positively controlled, although the induction fold in response to (R)−1,3-BDO was moderate at 5–10. This work provides a clear picture of (R)−1,3-BDO degradation in *P. putida* and offers potential (R)−1,3-BDO-responsive biosensors.

## RESULTS AND DISCUSSION

### Microbial screening for 1,3-BDO degradation

Five microorganisms, *Escherichia coli* W*, Klebsiella pneumoniae* J2B (17), *P. putida* KT2440*, Paracoccus denitrificans,* and *Vibrio natriegens*, were tested for their ability to grow on enantiopure (R)−1,3-BDO as the sole carbon source using a 96-well microplate for 25 h (Fig. S1). Glucose was used as a positive control, while a substrate-free medium served as a negative control. Among the strains tested, *P. putida* KT2440 demonstrated the most pronounced growth on (R)−1,3-BDO after a lag period before 15 h and was thus selected for further studies. The other strains showed marginal or no growth on (R)−1,3-BDO.

**Fig 1:**
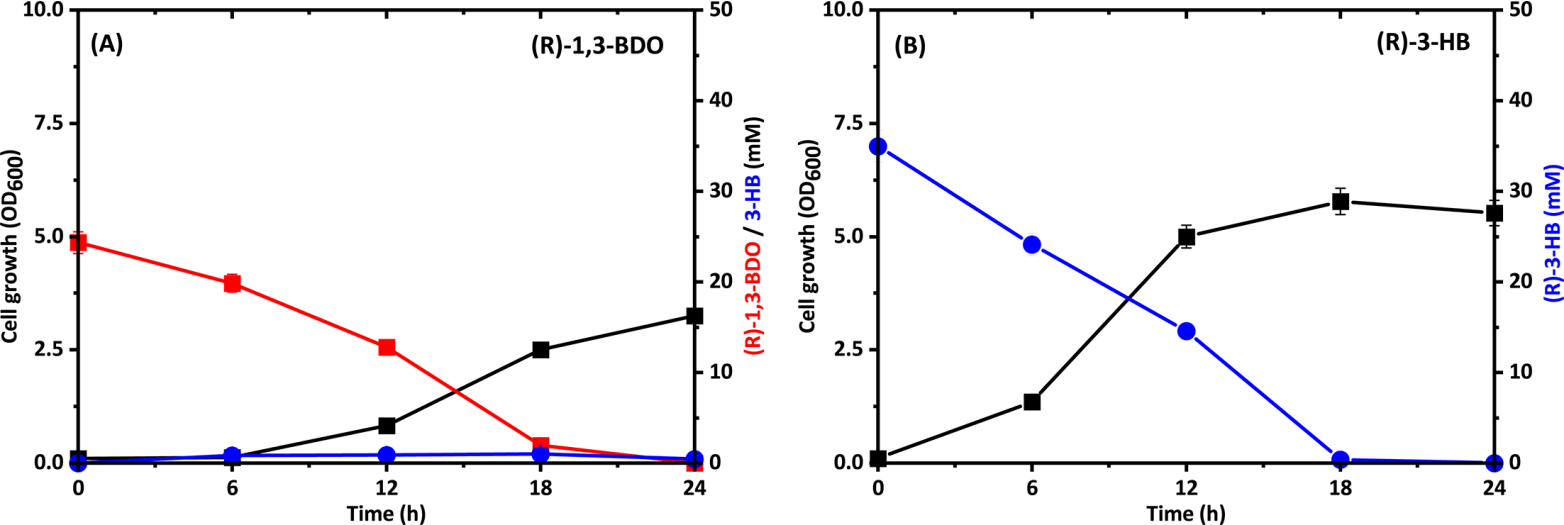
Growth and substrate utilization profile of *Pseudomonas putida* KT2440. (**A**) Cell growth, (R)−1,3-BDO consumption, and 3-HB accumulation. (**B**) Cell growth and (R)−3-HB consumption. Data are represented as mean ± standard deviation (SD) from triplicate experiments.

Flask growth experiments with *P. putida* were conducted under fully aerobic conditions with enantiopure (R)−1,3-BDO and (R)−3-HB, the latter of which is considered an intermediate appearing during the catabolism of the former (Fig. 1A and B). For both substrates, after a short lag period of <3–6 h, exponential growth was observed with the maximum specific growth rates (μ_max_) of 0.22 ± 0.02 h^−1^ for (R)−1,3-BDO and 0.40 ± 0.02 h^−1^ for (R)−3-HB. (R)−1,3-BDO added at 25 mM was completely consumed within 24 h, and (R)−3-HB added at 35 mM was consumed in 18 h, indicating that the acid is metabolized faster than the diol. However, the biomass yield for (R)−1,3-BDO and (R)−3-HB was the same at 0.49 ± 0.015 g/g. With (R)−1,3-BDO, accumulation of 3-HB, albeit at a low level, was detected, suggesting that 1,3-BDO is catabolized via 3-HB (18, 19). It was reported that the wild-type KT2440 grows on 1,4-BDO very slowly at a μ_max_ of 0.082 h^−1^, and after adaptive laboratory evolution (ALE), the growth rate could be enhanced to 0.33 h^−1^ (20). With (R)−1,3-BDO, even without ALE, a fast growth rate was observed, suggesting that (R)−1,3-BDO is a better substrate than 1,4-BDO.

### Transcriptomic analyses and catabolic pathways for (R)-1,3-BDO

**Fig 2:**
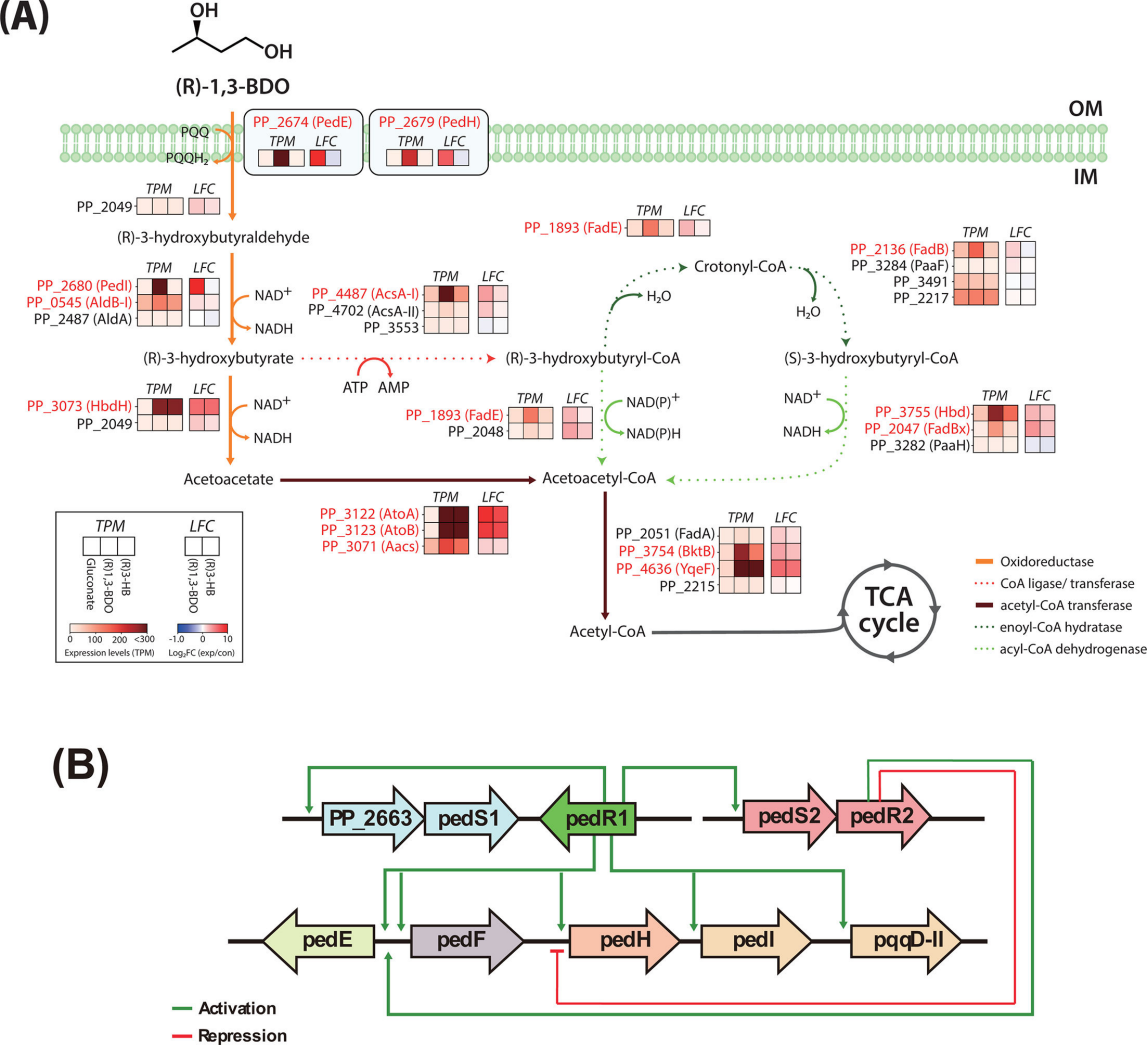
(**A**) Proposed catabolic pathways and transcriptome analysis for 1,3-BDO degradation in *P. putida* KT2440. Pathways were constructed based on transcript per million (TPM) and log fold change (LFC) values. Conversion of (R)−3-HB proceeds predominantly via acetoacetate, while an alternative route is predicted to contribute minimally and is therefore indicated by dotted lines. Enzyme classes are represented by the following color codes: oxidoreductases (orange); CoA ligases/transferases (red); enoyl-CoA hydratases (dark green); acyl-CoA dehydrogenases (light green); and acetyl-CoA transferases (brown). (**B**) Gene organization of the operons involved in (R)−1,3-BDO degradation and their regulation by PedR1 and PedR2. Arrows represent the operons that are positively regulated by PedR1 or PedR2 (green), while the blunt-ended red lines indicate genes negatively regulated by PedR2.

In a genome-wide fitness study using a transposon library (21), a catabolic route from mono- and di-alcohols to acetyl-CoA via corresponding acids was proposed. (R)−3-HB, which may be produced from (R)−1,3-BDO, appears as a ketone body in human metabolism and as a central intermediate released from intracellular poly(3-hydroxybutyrate) (PHB), a widespread microbial storage polymer; thus (R)−3-HB catabolism has been extensively investigated. However, because *P. putida* harbors multiple paralogs with overlapping annotations, purely sequence-based assignments remain incomplete. Here, we combine transcriptome profiling on (R)−1,3-BDO and (R)−3-HB with targeted gene deletion and enzyme activity measurements and propose a more refined (R)−1,3-BDO catabolic pathway (Fig. 2).

When (R)−1,3-BDO was supplied as the sole carbon source, strong transcriptional induction was observed for the PQQ-dependent alcohol dehydrogenase genes *pedE* (*PP_2674*) and *pedH* (*PP_2679*) (Fig. 2A; Fig. S2). Given their previously established roles in the oxidation of primary alcohols and diols (8, 20, 22), this expression pattern is consistent with their participation in the early steps of (R)−1,3-BDO oxidation. In contrast, *PP_2049*, encoding another predicted oxidoreductase, showed only modest induction under the same conditions (Fig. S2A). Transcripts corresponding to the NAD^+^-dependent aldehyde dehydrogenases *pedI* (*PP_2680*) and *aldB1* (*PP_0545*) were also elevated during growth on (R)−1,3-BDO, with *pedI* exhibiting the stronger response. Both enzymes have previously been associated with the metabolism of short-chain diols and related compounds (23). Their induction therefore supports the involvement of aldehyde oxidation reactions following initial (R)−1,3-BDO oxidation, although transcript levels alone do not establish reaction order or flux.

Downstream of (R)−3-HB formation, several routes for assimilation into central metabolism have been proposed. One such route involves oxidation of (R)−3-HB to acetoacetate (AA) and is attributed to *hbdH* (*PP_3073*). AA can subsequently be activated to acetoacetyl-CoA (AA-CoA) by CoA-ligase/transferase activities, notably *atoA*/*atoB* (*PP_3122*/*PP_3123*, which encode a two-subunit succinyl-CoA:3-oxoacid CoA transferase) and *aacS* (PP_3071). AA-CoA may then be cleaved to acetyl-CoA by thiolase-type enzymes such as *bktV* (*PP_3754*) and *yqeF* (*PP_4636*) (24–26). All these genes showed strong transcriptional induction during growth on (R)−1,3-BDO, consistent with their involvement in downstream assimilation. This coordinated transcriptional response suggests engagement of an AA-centered assimilation route under these conditions.

In parallel, transcripts for genes associated with alternative CoA-dependent routes were also enhanced. *acsA1* (*PP_4487*), which may catalyze CoA ligation of hydroxyacids, and *fadE* (*PP_1893*), encoding an acyl-CoA dehydrogenase, were upregulated during growth on (R)−1,3-BDO. These patterns indicate that *P. putida* has the genetic capacity to channel (R)−3-HB into metabolism through more than one downstream route, although transcriptional data alone cannot resolve the relative contribution of each pathway.

(R)−3-HB-CoA can be further routed through crotonyl-CoA and into the β-oxidation cycle. FadE (PP_1893), an acyl-CoA dehydrogenase homologous to *E. coli* FadE, catalyzes the formation of *trans*-2-enoyl-CoA intermediates (27). Hydration of crotonyl-CoA to (S)−3-HB-CoA and its oxidation to AA-CoA are catalyzed by the bifunctional FadB (PP_2136) and by additional hydratase/dehydrogenase paralogs: PP_3284 (PaaF), PP_3491, and PP_2217 (crotonase-like hydratases), and PP_3755 (Hbd), PP_2047 (FadBx), and PP_3282 (PaaH) (dehydrogenases). Our RNA-seq data showed induction of *PP_1893*, *PP_2136*, *PP_3755*, and *PP_2047* during growth on (R)−1,3-BDO; *PP_3491* and *PP_2217* were expressed at moderate baseline levels but were not further induced.

**Fig 3:**
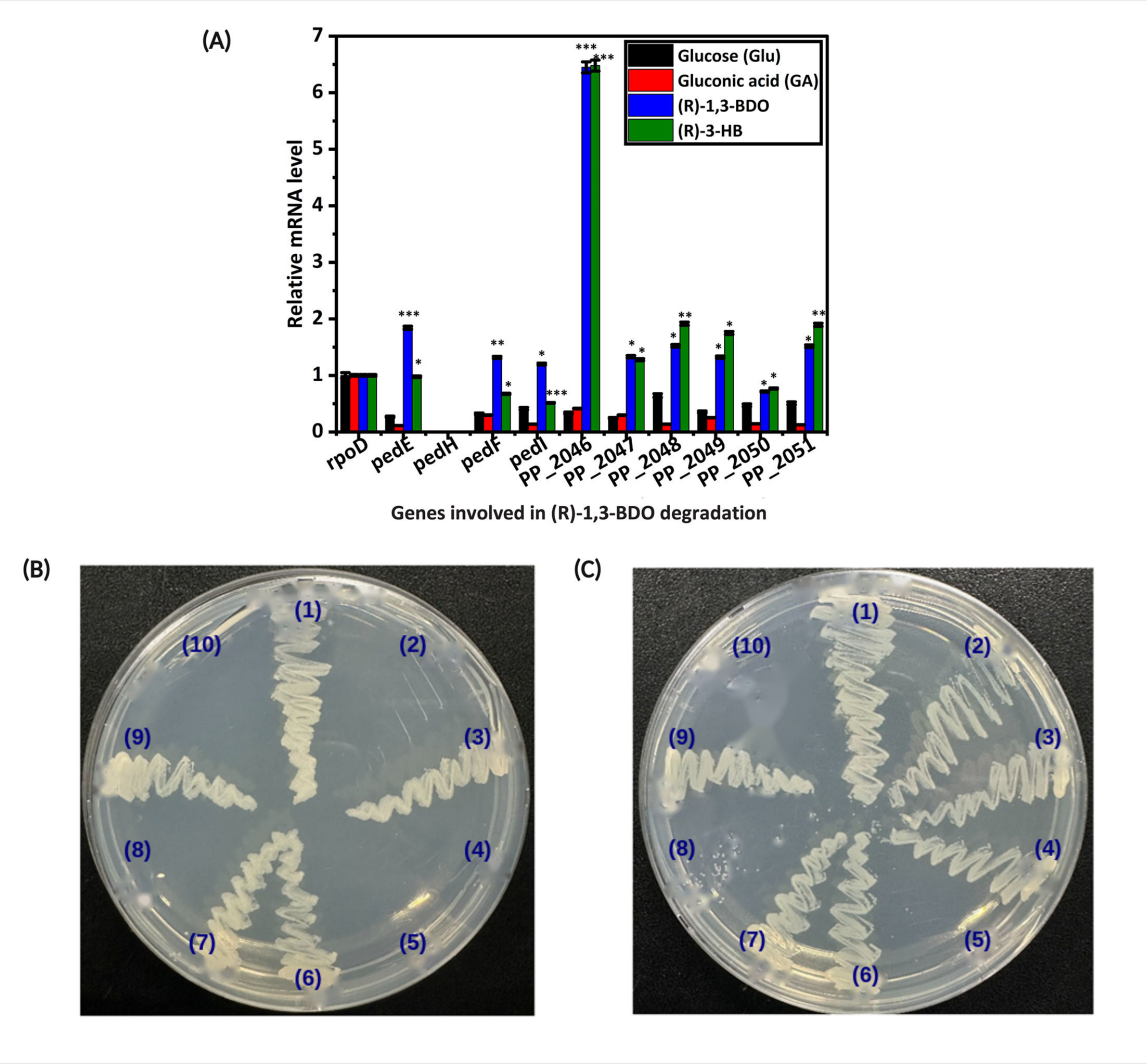
Experimental validation of genes involved in (R)−1,3-BDO degradation. (**A**) mRNA expression analysis for the candidate genes under different carbon sources: Glu, glucose; GA, gluconate; (R)−1,3-BDO, [(R)−1,3-butanediol], and (R)−3-HB, [(R)−3-hydroxybutyrate]. The error bars represent SD from three biological replicates. (**B** and **C**) Growth phenotypes of deletion mutants on (R)−1,3-BDO and (R)−3-HB, respectively. Strain designations: (1) WT, (2) Δ*pedE,* (3) Δ*pedI,* (4) Δ*pedE*-I, (5) Δ*PP_2046,* (6) Δ*PP_2047* (*fadBx*), (7) Δ*PP_2048,* (8) Δ*PP_2049,* (9) Δ*PP_2051,* and (10) Δ*PP_2047-51*.

Growth on (R)−3-HB resulted in a transcriptional profile largely overlapping with that observed on (R)−1,3-BDO, supporting the view that (R)−3-HB functions as a central intermediate in (R)−1,3-BDO assimilation. Notably, genes associated with the upstream oxidation of (R)−1,3-BDO (*pedE, pedH, pedI*, and *aldA*) were not induced during growth on (R)−3-HB, consistent with the interpretation that their expression responds specifically to the diol substrate rather than to (R)−3-HB itself. While differential induction was observed among genes associated with AA-based and CoA-based routes, these patterns are interpreted here as indicative of pathway availability rather than pathway dominance. Functional gene deletion analyses (Fig. 3) were therefore required to assess the essentiality of specific steps within this network.

Finally, transcriptional changes in known regulatory systems were consistent with previous studies of alcohol and diol metabolism in *P. putida* KT2440 (Fig. 2B; Fig. S2). Modest increases in transcript levels for *pedS1, pedR1*, *pedS2*, and *pedR2* were observed during growth on (R)−1,3-BDO, whereas no clear induction was detected during growth on (R)−3-HB. These observations support a regulatory response primarily linked to the presence of the diol substrate but do not directly define regulatory control points within the downstream catabolic network.

### Validation of the 1,3-BDO catabolic pathways by real-time PCR and gene deletion experiments

To further validate the (R)−1,3-BDO catabolic pathway, quantitative RT-PCR analysis was performed using cells grown on four different carbon sources, including glucose (Glu), gluconic acid (GA), (R)−1,3-BDO, and (R)−3-HB (Fig. 3A). The mRNA levels were normalized to the housekeeping gene *rpoD*. Both upstream (up to 3-HB) and downstream (after 3-HB) gene clusters exhibited substantial transcriptional upregulation in the cells grown on (R)−1,3-BDO or (R)−3-HB compared to glucose or gluconic acid. Notably, the upstream genes such as *pedE*, *pedF*, and *pedI* (but not *pedH*) were highly upregulated by (R)−1,3-BDO with a higher fold change than by (R)−3-HB. Similarly, the downstream gene clusters, *PP_2046* and *PP_2047–2051*, were also highly expressed, more so by (R)−3-HB. *PP_2046*, encoding the LysR-type transcriptional regulator (LTTR), which controls the expression of the downstream operon (*PP_2047–2051*), showed strong induction (>15-fold) in the presence of (R)−1,3-BDO or (R)−3-HB. Although the transcription patterns between RNA-seq and RT-PCR were generally similar, several differences were observed. First, in the RNA-seq, the upstream genes such as *pedE*, *pedH,* and *pedI* were highly expressed only with 1,3-BDO and not at all with 3-HB. In comparison, in RT-PCR, those genes, except *pedH* (not upregulated at all), were upregulated by both (R)−1,3-BDO and (R)−3-HB (although to a lesser extent). Second, in RNA-seq, the fold changes of upregulation were generally much greater. Despite these differences, both analyses agree that the *ped* clusters are highly upregulated when (R)−1,3-BDO is used as a carbon source. This suggests that the *ped* clusters are responsible for the catabolism of (R)−1,3-BDO, like other diols such as 1,4-BDO and ethylene glycol (20, 23, 28).

To assess gene functions and essentiality, deletion mutants were constructed and evaluated for growth on (R)−1,3-BDO and (R)−3-HB by agar plate (Fig. 3B and C) and liquid culture (Fig. S3). Deletion of *pedE* resulted in a complete loss of growth on (R)−1,3-BDO, indicating that PedE is indispensable for the initial oxidation step of (R)−1,3-BDO. In comparison, the Δ*pedI* mutant exhibited similar growth on (R)−1,3-BDO to the wild type, implying functional redundancy among aldehyde dehydrogenases in *P. putida* KT2440. PedE has been reported to have broad substrate specificity for various alcohols such as ethanol, 1-butanol, and 1,4-BDO (8, 11, 29, 30), and the current results indicate that its activity extends to (R)−1,3-BDO. Among the downstream genes, *PP_2046* was observed to be essential; its deletion abolished growth on both (R)−1,3-BDO and (R)−3-HB. This suggests that without this regulator, expression of the *PP_2047-2051* operon, especially *PP_2049* (see below), is too low to support the utilization of (R)−3-HB. Among the genes in the *PP_2047-2051* operon, only the Δ*PP_2049* mutant lost the ability to grow on (R)−1,3-BDO as well as (R)−3-HB; other knockout strains lacking *PP_2047* (3-hydroxyacyl-CoA dehydrogenase), *PP_2048* (acyl-CoA dehydrogenase), or *PP_2051* (3-ketoacyl-CoA thiolase) demonstrated normal growth on (R)−1,3-BDO and (R)−3-HB (Fig. 3B and C; Fig. S3). This suggests that the growth defect observed in the mutants Δ*PP_2046* and Δ*PP_2047-2051* is attributable to the loss of PP_2049.

The essential role of PP_2049 in the catabolism of (R)−1,3-BDO and (R)−3-HB warrants further clarification. Many previous studies have indicated that instead of *PP_2049*, *PP_3073*, encoding β-hydroxybutyrate dehydrogenase (HbdH), is essential for growth on (R)−3-HB. This NAD^+^-dependent HbdH, belonging to the short-chain dehydrogenases/reductases (SDRs) family, can be highly active (for example, the enzyme from *P. putida* ZIMET 10947, GenBank accession number AJ310211.2, had an activity of~3,100U/mg and a *K_m_* of ~1.5mM (24)) and is (R)-specific. Fitness (RB TnSeq) data in KT2440 have also demonstrated a strong negative fitness defect for the *PP_3073* disruption when cells grew on racemic 1,3-BDO (21). Furthermore, in *P. aeruginosa* (having a similar pathway), *hbdH* (also named as *bdhA*) and its σ^54^/EBP regulator were required for growth on (R)−3-HB (31). In our RNA-seq studies, *PP_3073* was also highly expressed, supporting its important role in the growth on (R)−1,3-BDO or (R)−3-HB. However, our gene deletion experiments indicate that PP_2049 is essential even in the presence of PP_3073.

**Fig 4:**
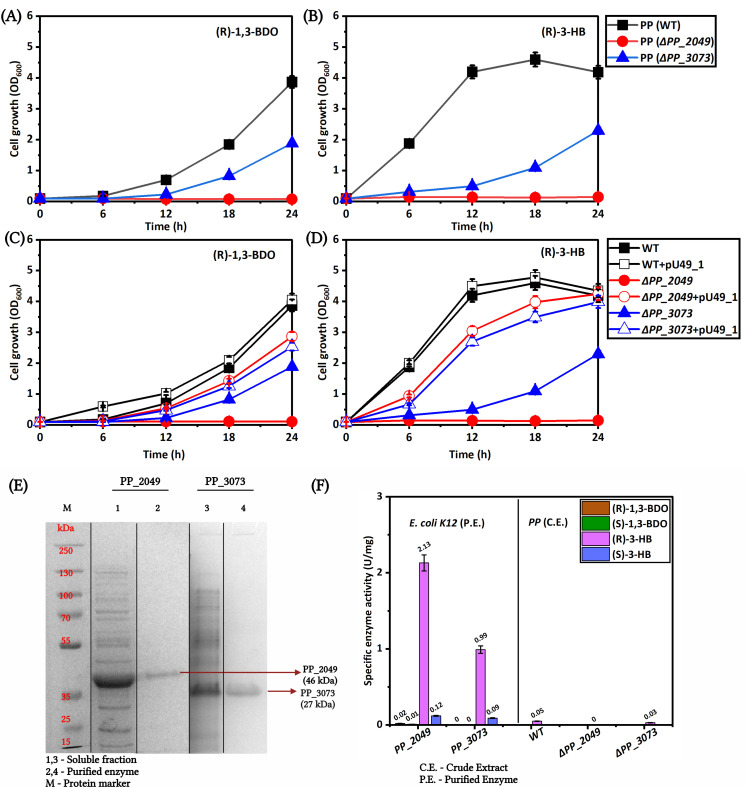
Functional analysis of PP_2049 and PP_3073 in (R)−1,3-BDO catabolism. (**A**) Growth of WT, Δ*PP_2049*, and Δ*PP_3073* strains on (R)−1,3-BDO. (**B**) Growth of the same strains on (R)−3-HB. (**C**) Growth of WT, ΔPP_2049, and ΔPP_3073 on (R)−1,3-BDO with or without overexpression of PP_2049 from plasmid pU49_1. (**D**) Growth of the same strains on (R)−3-HB under identical conditions. (**E**) SDS-PAGE of the His-tagged purified PP_2049 and PP_3073, with individual protein bands separated by thin black lines for clarity. (**F**) Enzyme activity of purified PP_2049 and PP_3073 expressed in *E. coli* K12 (MG1655) towards (R)−1,3-BDO, (S)−1,3-BDO, (R)−3-HB, and (S)−3-HB, along with the crude enzyme activity of PP (WT), Δ*PP_2049*, and Δ*PP_3073* strains toward (R)−3-HB. Data are presented as mean ± standard deviation (SD) from three independent biological replicates.

To clarify the roles of PP_2049 and PP_3073, further experiments were conducted. First, *PP_3073* was deleted, and the mutant was tested for growth on (R)−1,3-BDO and (R)−3-HB in liquid cultures (Fig. 4A and B); the mutant could grow on both substrates, although at reduced rates. In contrast, the Δ*PP_2049* mutant could not grow at all, confirming its essentiality. To further validate the function of PP_2049, a complementation experiment was performed by expressing *PP_2049* from a plasmid, pU49_1 (Fig. 4C and D). Overexpression restored and enhanced growth on both (R)−1,3-BDO and (R)−3-HB in all tested backgrounds, including the wild-type, Δ*PP_2049*, and Δ*PP_3073* strains. Furthermore, when the expression level of *PP_2049* was modulated by varying the untranslated region (UTR) in the expression plasmid, a dose-dependent relationship between the level of episomal *PP_2049* expression and cell growth on (R)−3-HB was observed in the Δ*PP_2049* strain (Fig. S4A and B). Collectively, these results suggest that PP_2049 is essential for the growth of KT2440 on (R)−3-HB, whereas PP_3073 is not essential and cannot replace the function of PP_2049 in its absence.

To further explore the roles of PP_2049 and PP_3073, *in vitro* enzyme activities of His-tagged recombinant PP_2049 and PP_3073 were measured with NAD^+^ as cofactor (Fig. 4E and F). PP_2049 exhibited a specific activity of 2.13 U/mg protein on (R)−3-HB, whereas PP_3073 showed 0.99 U/mg protein, indicating that PP_2049 has ~2-fold higher activity than PP_3073. During the activity measurement, equimolar NADH and acetoacetate (AA) were generated for both PP_2049 and PP_3073, indicating that these enzymes convert (R)−3-HB to AA with NAD^+^ as cofactor. Both enzymes showed minor activity toward (S)−3-HB (about 1/12th–1/18th of that toward (R)−3-HB), confirming that they are highly (R)-specific. In addition, with (R)−1,3-BDO or (S)−1,3-BDO, they did not show any activity, indicating that they are not involved in the oxidation of those diols. The dehydrogenase activity on (R)−3-HB was also determined using crude cell extracts of the wild-type, Δ*PP_2049*, and Δ*PP_3073* (Fig. 4F). The wild-type and Δ*PP_3073* showed detectable activity at ~0.05 U/mg and ~0.03 U/mg, respectively, while Δ*PP_2049* did not show any detectable activity. This suggests that, although *PP_3073* is highly transcribed (Fig. 2) and its specific activity as a purified enzyme is not negligible, its contribution to (R)−3-HB oxidation in the wild-type cell is insufficient to support growth.

It is worth noting that in our study, PP_3073 (AAN68681) from *P. putida* KT2440 exhibited a specific activity of only 0.99 U/mg protein on (R)−3-HB, which is more than three orders of magnitude lower than the ~3,100U/mg reported for BdhA (AJ310211.2) from a different *P. putida* strain, ZIMET 10947 (24). Even PP_2049, which showed higher activity than PP_3073 at 2.13 U/mg protein, remains far below the ZIMET 10947 enzyme. Pairwise sequence comparison revealed 94.9% amino acid identity between PP_3073 and the ZIMET 10947 enzyme (Fig. S5), indicating that only ~13 residues differ over the 256-amino-acid sequence. To place this discrepancy in a broader context, we compiled published kinetic data for bacterial HBDHs from several species (Table S1). The reported specific activities span a wide range, from ~1.8U/mg for *Rhodopseudomonas spheroides* (32) to ~3,100U/mg for *P. putida* ZIMET 10947, with PP_3073 from KT2440 (0.99 U/mg) at the lowest end. Among the characterized enzymes, HBDH from *Pseudomonas fragi* (BAD86668), whose X-ray crystal structure has been solved (26), is notable for its high activity (~640U/mg), approximately one-fifth that of the ZIMET 10947 enzyme, despite sharing only 59.4% amino acid identity with it.

Importantly, all four critical substrate-binding residues identified by Feller et al. (24)—Gln91, His141, Lys149, and Gln193 (ZIMET numbering)—as well as the entire catalytic tetrad (Asn111, Ser139, Tyr152, and Lys156) characteristic of the SDR family, are strictly conserved across the ZIMET 10947, KT2440, and *P. fragi* enzymes (Fig. S5). This rules out direct active-site mutations as the cause of the observed activity differences. Instead, the sequence divergence is concentrated in the substrate-binding loop (α1–α2, corresponding to loop 3 (24)), which is the most variable region in multiple-sequence alignments of bacterial HBDHs. In the X-ray crystal structure of *P. fragi* HBDH, the only bacterial HBDH for which a high-resolution crystal structure is available, this loop was completely disordered (missing electron density for 24 residues, Pro191–Glu214) in the NAD^+^ complex, demonstrating its high intrinsic mobility (26). This loop (i) undergoes a critical open-to-closed conformational change upon substrate binding (24, 25) and (ii) contains second-shell residues that cover the active site in the closed conformation and help exclude solvent, a step that is often rate-limiting in SDR enzyme catalysis (24). Consistent with the functional importance of this region, Machado et al. (33, 34) demonstrated that a single amino acid difference in this region—His150 in *Psychrobacter arcticus* HBDH versus Asn145 in *Acinetobacter baumannii* HBDH—accounts for a large part of the kinetic difference between those two orthologs, with the His→Ala mutation causing a 55-fold decrease in *k_cat_*. To visualize the structural consequences of the ~13 residue differences between PP_3073 (KT2440) and BdhA (ZIMET 10947), we generated AlphaFold-predicted structures for both enzymes and superimposed them (Fig. S6). Several of the differing residues are located within or adjacent to the three substrate-binding loop regions (loops 1–3), where they may influence substrate positioning, loop dynamics, or the rate of the open-to-closed conformational transition, thereby contributing to the large difference in catalytic activity.

Taken together, these results indicate that PP_2049 is the primary enzyme responsible for the conversion of (R)−3-HB to AA, with PP_3073 playing a supporting role. Our results indicate that (R)−3-HB is catabolized predominantly via AA rather than via (R)−3-HB-CoA, although the enzymes channeling (R)−3-HB to (R)−3-HB-CoA and/or crotonyl-CoA were also upregulated according to the RNA-seq experiments.

### Regulation of (R)-1,3-BDO degradation pathways

Regulation of the *ped* operons has been investigated in several strains, including *P. putida*, *P. aeruginosa*, and *E. coli* (10, 12, 21) (Fig. 2B). These studies provide a useful framework, but we noticed that establishing (R)−1,3-BDO biosensors requires (i) confirming that the cascade by PedR1 and PedR2 is activated by (R)−1,3-BDO, (ii) determining whether (R)−1,3-BDO-responsive promoters require PedR2 alone or PedR1 together with PedR2, and (iii) identifying the actual inducer of the cascade—(R)−1,3-BDO, its oxidation product (R)−3-HB, or both.

**Fig 5:**
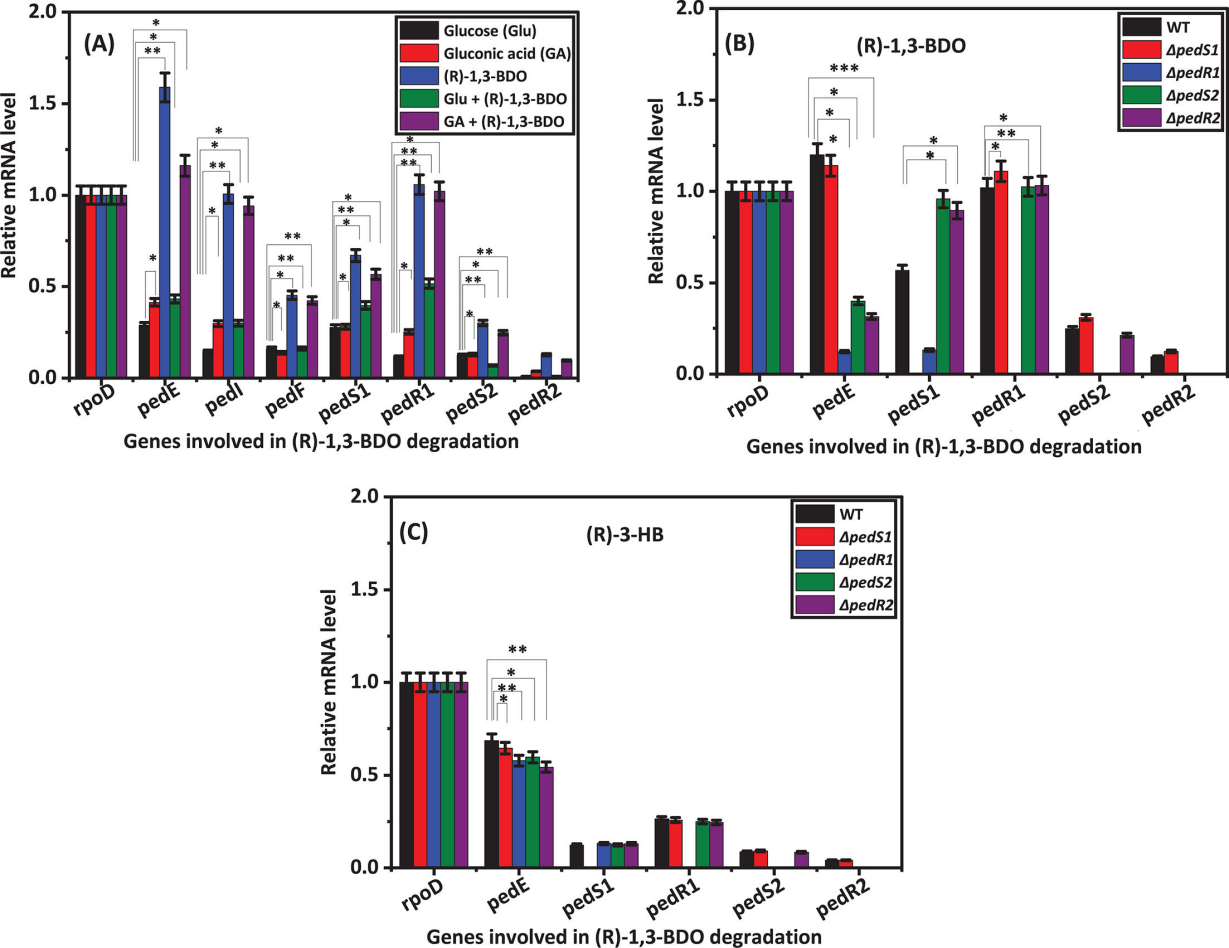
Impact of carbon source and transcription factor deletion on (R)−1,3-BDO degradation gene expression. (**A**) mRNA profiling of degradation pathway genes and regulatory genes with different substrates [Glu; glucose, GA; gluconic acid, and (R)−1,3-BDO], normalized to (R)−1,3-BDO. (**B**) Gene expression in transcription factor mutants (WT, Δ*pedR1,* Δ*pedS2,* and Δ*pedR2*) for *pedE, pedI, pedF, pedR1, pedS2, pedR2,* and *pedS1* with gluconic acid and (R)−1,3-BDO. (**C**) Gene expression in the same transcription factor mutants for the same gene set with gluconic acid and (R)−3-HB. All results are shown with SD from triplicate experiments.

To analyze these regulatory systems, cells must be cultured on a carbon source that supports growth of regulatory-gene deletion mutants, because disruption of positive regulators can abolish growth on (R)−1,3-BDO. The choice of carbon source is also critical because strong carbon catabolite repression (CCR) can mask induction by 1,3-BDO or 3-HB. Among the carbon sources tested, gluconate supported growth without causing CCR (Fig. 5; Fig. S7). In the presence of gluconate, (R)−1,3-BDO markedly upregulated transcription of most *ped* genes, including catabolic (*pedE*, *pedH*, *pedI*, and *pedF*) and regulatory (*pedS1*, *pedR1*, *pedS2*, and *pedR2*) genes, with expression levels only slightly lower than when (R)−1,3-BDO served as the sole carbon source. In contrast, glucose strongly repressed these genes, reducing their expression in many cases to near basal (non-induced) levels (Fig. 5A). Consistent with these transcriptional patterns, (R)−1,3-BDO was co-consumed with gluconate but not with glucose (Fig. S7).

Using gluconate as the growth substrate and either (R)−1,3-BDO (Fig. 5B) or (R)−3-HB (Fig. 5C) as the inducer, we next examined the roles of *pedS1*, *pedR1*, *pedS2*, and *pedR2* in regulating *ped* transcription. With (R)−1,3-BDO, transcription of catabolic genes (*pedE*, *pedF*, and *pedI*) was strongly reduced in the Δ*pedR1* mutant and moderately reduced in Δ*pedS2* and Δ*pedR2*, whereas no such effect was observed in Δ*pedS1*. Expression of *pedS2R2* was also markedly decreased in Δ*pedR1*. In contrast, (R)−3-HB did not significantly induce any of the catabolic or regulatory genes. Growth experiments with (R)−1,3-BDO or (R)−3-HB as the sole carbon source gave consistent results: with (R)−1,3-BDO, Δ*pedR1* completely lost the ability to grow, whereas Δ*pedR2* and Δ*pedS2* still grew, albeit more slowly, and Δ*pedS1* grew unaffected (Fig. S8). When (R)−3-HB was used as the sole carbon source, all strains grew well and similarly.

Taken together, these data support the regulatory scheme in Fig. S9 and lead to the following conclusions. First, catabolic genes such as *pedE*, *pedI*, and *pedF* are under positive control of both PedR1 and PedR2. Second, the regulatory operon *pedS2R2* is positively controlled by PedR1. Third, the primary signaling molecule for this regulatory cascade is (R)−1,3-BDO (or its aldehyde intermediate), not (R)−3-HB. These conclusions are in good agreement with previous studies on other alcohols, which showed that PedR1 acts as a master positive transcriptional regulator of both catabolic (*pedE*, *pedI*, and *pedF*) and regulatory (*pedS1*, *pedS2*, and *pedR2*) genes. It has been reported that in *A. baumannii*, deleting *emaSR* (corresponding to *pedS1R1*) abolishes the strain’s ability to grow on ethanol as a carbon source, underlining that no other cytosolic sensors are available and the two-component route has evolved to control ethanol/acetate utilization (35).

Importantly, our results challenge the widely accepted model that PedR1, as a master regulator, controls catabolic genes solely and indirectly through PedR2 (11, 23). If this indirect route were the sole mechanism, transcript levels of catabolic genes should be more strongly reduced in Δ*pedR2* than in Δ*pedR1*, and Δ*pedR2* should completely lose the ability to grow on (R)−1,3-BDO. However, our data show the opposite: catabolic gene expression was most severely impaired in Δ*pedR1*, not in Δ*pedR2*, and only Δ*pedR1* was unable to grow on (R)−1,3-BDO as the sole carbon source (Fig. S8). These findings strongly suggest that PedR1 also directly activates catabolic genes in *P. putida* KT2440, independently of PedR2. To date, such direct regulation by PedR1 has been reported only once, in *P. putida* HK5 (12), and our results in KT2440 provide the second line of evidence supporting this regulatory architecture.

Another notable finding is that PedS1, the putative sensor kinase of the PedS1/PedR1 two-component system, is dispensable for PedR1-dependent activation. Deletion of *pedS1* had no detectable effect on the expression of catabolic genes or on growth with (R)−1,3-BDO as the sole carbon source, indicating that PedR1 can be activated through an alternative, yet unidentified, sensor kinase. This observation is consistent with findings in *P. aeruginosa*, where deletion of *ercS* (the *pedS1* homolog) does not abolish promoter activity as long as *agmR* (the *pedR1* homolog) is present (11). The existence of such an alternative activation route implies that the signaling network controlling (R)−1,3-BDO catabolism is more complex than the simple linear PedS1→PedR1→PedS2R2 cascade currently assumed, and identifying this unknown sensor kinase will be an important objective for future studies (11, 36).

### Development of (R)-1,3-BDO-responsive biosensor system

**Fig 6:**
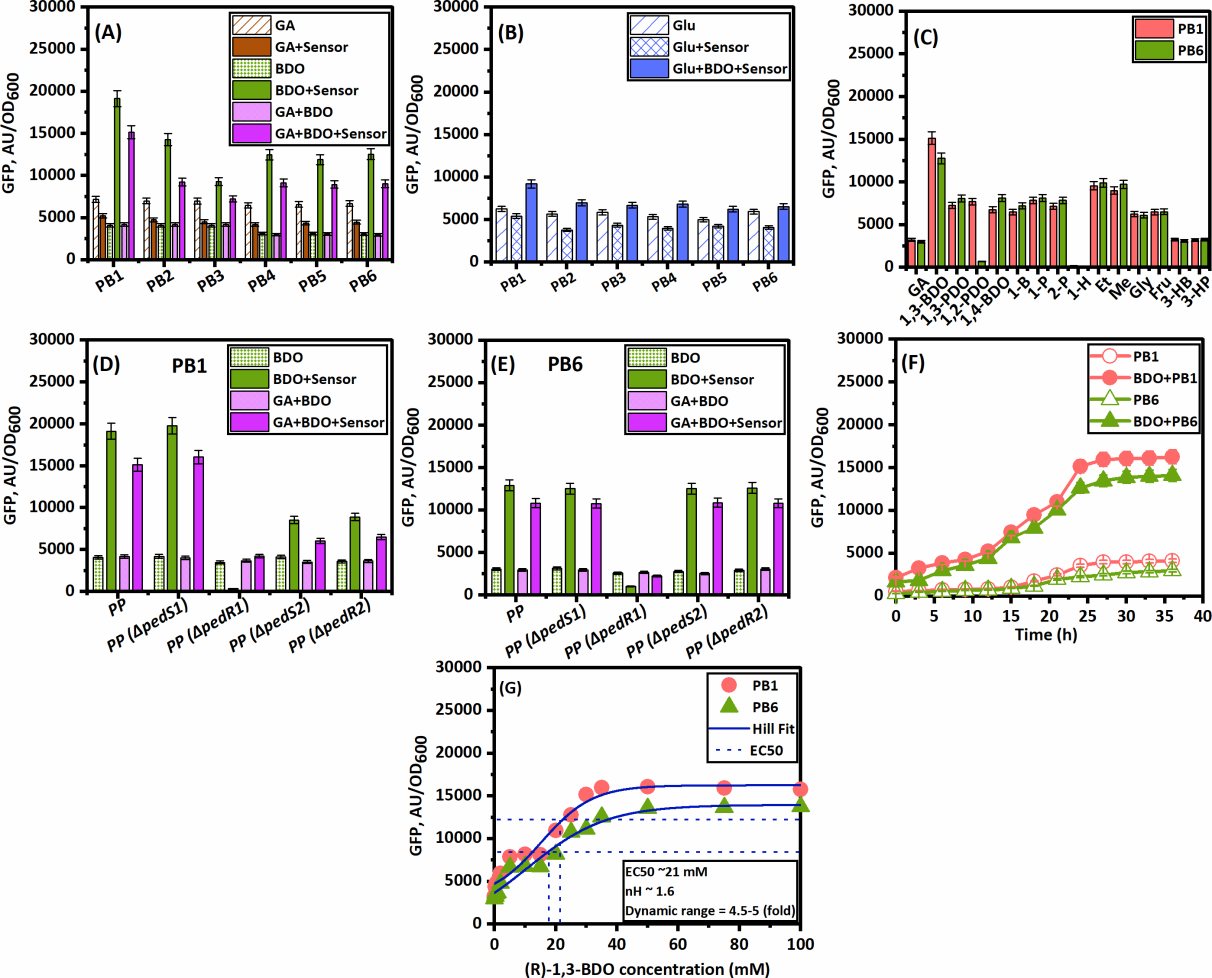
GFP-based biosensor performance using promoter-reporter constructs. (**A**) GFP fluorescence response of strain PP (WT) carrying promoter-*gfp* fusion constructs—PB1 (*pedE* promoter), PB2 (*pedF* promoter), PB3 (*pedI* promoter), PB4 (*pedS1* promoter), PB5 (*pedR1* promoter), and PB6 (*pedS2/R2* promoter) - grown on gluconic acid (GA) as the carbon source with (R)−1,3-BDO as the inducer. (**B**) Corresponding GFP fluorescence response of the same constructs grown on Glu (glucose) as the carbon source with (R)−1,3-BDO as the inducer. (**C**) GFP fluorescence response of PB1 and PB6 to 35 mM of various structurally similar compounds. (**D**) Effect of regulatory component deletions on PB1 biosensor performance. (**E**) Effect of regulatory component deletions on PB6 biosensor performance. (**F**) Time-course fluorescence profiles of PB1 and PB6 in PP (WT). (**G**) Dose-dependent induction and corresponding Hill plots representing the EC_50_, Hill coefficient (nH), and dynamic range for the PB1 and PB6. Fluorescence is shown as AU/OD₆₀₀ (arbitrary units normalized to optical density at 600 nm). Error bars indicate SD calculated from triplicate. Abbreviations: GA, gluconic acid; 1,3-BDO, (R)−1,3-butanediol; 1,3-PDO, 1,3-propanediol; 1,2-PDO, 1,2-propanediol; 1,4, BDO-1,4-butanediol; 1-B, 1-butanol; 1-P, 1-propanol; 2-P, 2-propanol; 1-H, 1-hexanol; Et, ethanol; Me, methanol; Gly, glycerol; Fru, fructose; 3-HB, (R)−3-hydroxybutyrate; 3-HP, 3-hydroxypropionic acid.

To develop (R)−1,3-BDO-responsive biosensors, six promoters—P*pedE*, P*pedF*, P*pedI*, P*pedS1*, P*pedR1*, and P*pedS2R2*—were evaluated by placing *gfp* downstream of each promoter and measuring fluorescence in *P. putida* (Fig. 6). All promoters were inducible by (R)−1,3-BDO when provided alone or together with gluconate (~20% less), whereas little or no induction was observed in the presence of glucose, confirming strong catabolite repression (Fig. 6A and B). Among the promoters tested, P*_pedE_* (designated PB1) showed the highest dynamic range (~6-fold induction), and the regulatory promoters P*_pedS1_*, P*_pedR1_*, and P*_pedS2R2_* (designated PB4, PB5, and PB6, respectively) showed similar but slightly weaker responses (~5-fold), with comparable basal activities. These single-digit-fold changes are in agreement with most two-component systems reported in bacteria (35, 37).

Inducer specificity was examined for PB1 (P*_pedE_*) and PB6 (P*_pedS2R2_*). A panel of diols, mono- and tri-alcohols, fructose, and β-hydroxy acids [(R)−3-HB and 3-HP] (35 mM each) was tested (Fig. 6C). For both promoters, (R)−1,3-BDO elicited the strongest response, followed by ethanol and methanol. Most other compounds gave a modest ~2.5-fold induction, except 1-hexanol, which showed little or no effect, likely owing to its low solubility and toxicity. Notably, the β-hydroxy acids did not activate P*_pedE_*, despite causing weak upregulation of *ped* transcripts in the RT-PCR analyses (Fig. 3A and 5).

To define the regulatory requirements of PB1 and PB6, each construct was introduced into Δ*pedR1*, Δ*pedS2*, Δ*pedR2*, and Δ*pedS1* mutants, and GFP induction by (R)−1,3-BDO was measured (Fig. 6D and E). For PB1, induction was abolished in Δ*pedR1* and reduced but still detectable in Δ*pedS2* and Δ*pedR2*, whereas Δ*pedS1* behaved like the wild type. In comparison, for PB6, induction was lost only in Δ*pedR1* and was unaffected by the other deletions. These results indicate that P*_pedE_* requires PedR1, PedS2, and PedR2, whereas P*_pedS2R2_* depends solely on PedR1. These observations are also consistent with the findings described above that PedR1 regulates P*_pedE_* both directly and indirectly via PedS2R2.

Biosensors PB1 and PB6 were further characterized for response time, EC_50_, and dynamic range using gluconate as the carbon source. In 96-well microplate cultures, maximum induction was reached within 25–30 h after (R)−1,3-BDO addition for both PB1 and PB6 (Fig. 6F). This response time was shortened to~20h (along with 3–4 times higher specific GFP) in fully aerobic flask cultures (Fig. S10B), likely reflecting the faster and higher growth under more aerobic conditions: the OD plateau (~10OD in flask vs. ~1.5OD in microplate) was reached at~15h in flask cultures compared to~20h in microplate cultures (Fig. S10A and C). Dose-response experiments revealed increasing fluorescence with rising (R)−1,3-BDO concentrations up to~35mM for both sensors (Fig. 6G). Hill equation fitting yielded EC_50_ values of~21mM, dynamic ranges of 4.5–5.0, and Hill coefficients of ~1.6.

**Fig 7:**
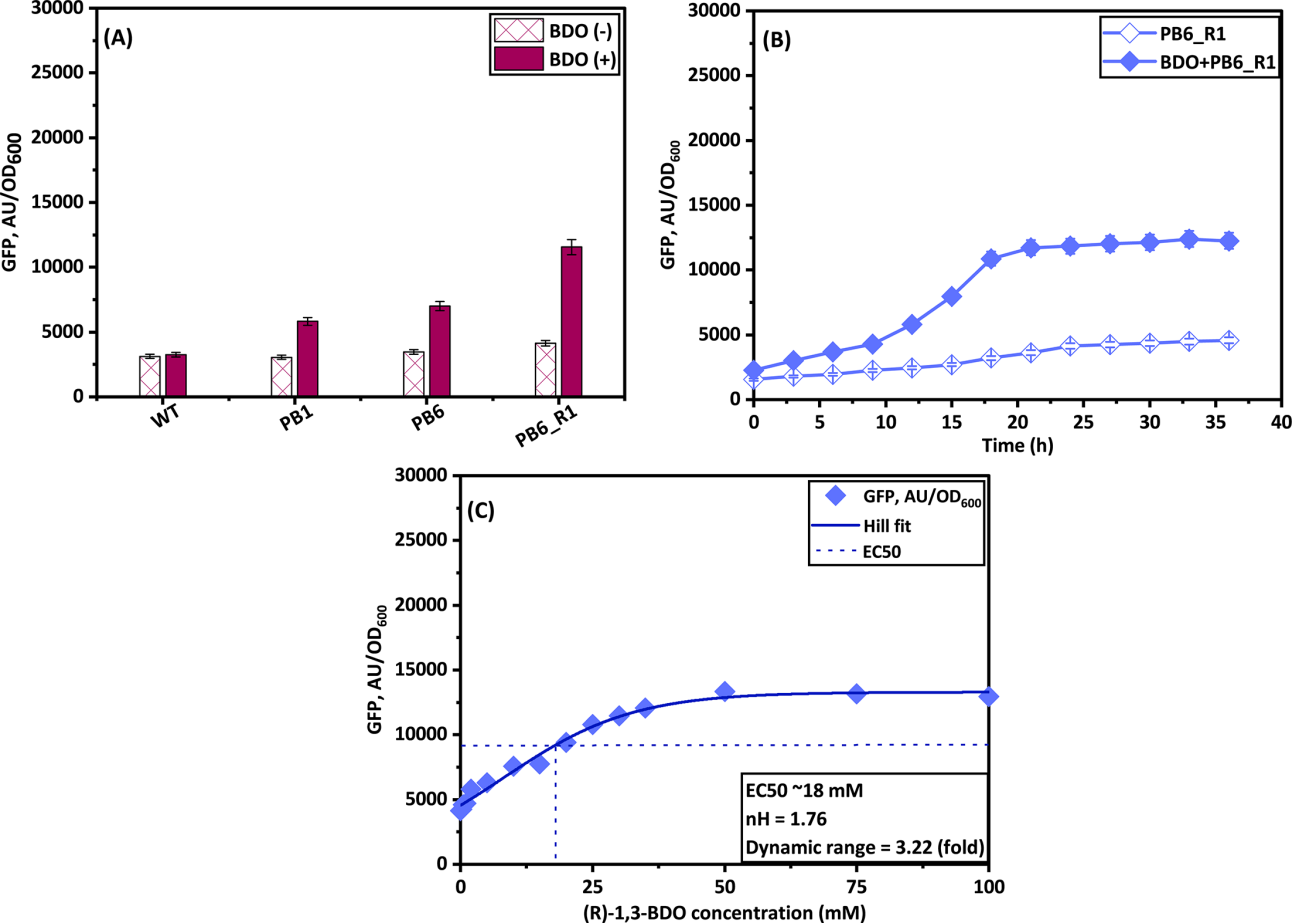
Portability of *pedE* (PB1) and *pedS2/R2* (PB6) promoter-based biosensors in *E. coli* K-12. (**A**) Functional analysis of PB1, PB6, and PB6_R1 (PB6 co-expressed with *pedR1* response regulator). (**B**) Time-course fluorescence profiles of PB6_R1. (**C**) Dose-dependent induction and corresponding Hill plots for PB6_R1. - indicates biosensor strains without inducer, and + indicates strains carrying the respective plasmid construct in the presence of (R)−1,3-BDO. All assays were performed in triplicate; error bars represent SD.

The two sensor constructs were then tested in *E. coli* K-12 to assess portability (Fig. 7). For PB6, a variant (PedR1–P*_pedS2R2_–gfp*; designated PB6_R1) with constitutive *pedR1* expression under the *zwf* promoter was also examined. All three sensors responded to (R)−1,3-BDO, but PB6_R1 showed the highest response (~3-fold induction), indicating that P*_pedS2R2_* functions as an effective sensor in *E. coli* K-12 when PedR1 is supplied (Fig. 7A). This also suggests that *E. coli* K-12 may harbor an endogenous sensor kinase capable of activating PedR1, albeit less efficiently than in *P. putida*; identification of this putative kinase warrants further investigation. Maximum induction was reached within ~20 h in microplate cultures, slightly earlier than in *P. putida*, likely reflecting the faster growth rate of *E. coli* (Fig. 7B). Dose-response analysis by Hill equation showed increasing fluorescence up to 50 mM (R)−1,3-BDO, with an EC_50_ of ~18 mM, which are similar to the case in the native *P. putida* (Fig. 7C).

The (R)−1,3-BDO-responsive promoters characterized here, particularly P*_pedE_* and P*_pedS2R2_*, provide a useful starting point for biosensing and dynamic pathway regulation, offering robust induction and clear dose-response behavior. However, their current utility is limited by partial cross-reactivity with other short-chain alcohols, sensitivity to carbon catabolite repression, and dependence on native regulators (PedR1 and the PedS2-PedR2 two-component system), which constrain portability and modular use in heterologous hosts. Future efforts to increase ligand specificity, reduce CCR sensitivity, and minimize the number of required regulatory components—through rational promoter redesign, directed evolution, or synthetic transcription factor engineering—should yield BDO biosensors with expanded dynamic range, tunable thresholds, and improved orthogonality suitable for *in vivo* screening and closed-loop pathway control (37).

### Conclusion

This study establishes a coherent catabolic and regulatory framework for (R)−1,3-butanediol degradation in *P. putida* KT2440 and translates these insights into biosensor development. (R)−1,3-BDO is oxidized via the *ped* cluster to (R)−3-HB and further funneled through the LysR-controlled PP_2047–2051 operon to acetoacetate and acetyl-CoA, with *pedE* and PP_2049 serving as essential enzymes for the upstream and downstream steps, respectively. Notably, PP_2049 was found to be more critical than the canonical HbdH (PP_3073) for (R)−3-HB oxidation. Regulatory analysis revealed that PedR1 directly activates catabolic genes independently of PedR2—challenging the prevailing indirect-only model—while PedS1 is dispensable, implying the existence of an alternative sensor kinase. Based on this regulatory circuitry, GFP-based biosensors driven by the *pedE* and *pedS2R2* promoters were constructed. Although portability of the *pedE* biosensor is constrained by its dependence on the complete PedR1–PedS2–PedR2 cascade, the PedR1-driven *pedS2R2* module (PB6_R1) functioned robustly in both *P. putida* and *E. coli*, highlighting its potential as a portable (R)−1,3-BDO-responsive element for biosensing and dynamic pathway regulation.

## MATERIALS AND METHODS

### Materials

Plasmid DNA purification and PCR gel purification kits were obtained from Cosmogenetech (Seoul, Korea). A total RNA isolation kit was bought from Macherey-Nagel, Germany. Unless specified otherwise, all other chemicals and reagents used in this study were procured from Sigma-Aldrich (St. Louis, MO, USA).

### Plasmid construction and cloning strategy

Table 1 provides the list of bacterial strains and plasmids used in this study. For routine cloning and plasmid maintenance, the *E. coli* Top10 strain was used. Chromosomal gene deletions in *P. putida* were done using the in-frame tagged deletion/insertion method (38). The P*_pedE_*, P*_pedF_,*, P*_pedI,_* P*_pedS1,_* P*_pedR1_,* and P*_pedS2/R2_* were amplified from the genomic DNA of *P. putida*, while the *gfp* gene, which encodes green fluorescence protein (GFP), was amplified from pUCPK_P*zwf*_*gfp*. The fragments were cloned into the pUCPK vector and subsequently confirmed by sequencing (Macrogen, Korea). The resulting constructs were then transformed into *P. putida*, and the deletion mutant strains.

**TABLE 1:** Strains and plasmids used in this study

Strain or plasmid	Description	Source
Strains		
*E. coli* Top10	Cloning host	Invitrogen
*Pseudomonas putida* KT2440 (PP)	Wild type	Lab strain
*E. coli* W ATCC 9637	Wild type	Lab strain
*Klebsiella pneumoniae* J2B	Wild type	Lab strain
*Pseudomonas denitrificans* ATCC 13867	Wild type	Lab strain
*Vibrio natriegens* ATCC 14048	Wild type	KCTC
*E. coli* K12 (MG1655)	Wild type	Lab strain
Δ*pedE*	Deletion of *pedE* in PP	This study
Δ*pedI*	Deletion of *pedI* in PP	This study
Δ*pedE-I*	Deletion of *pedE-I* in PP	This study
ΔPP_2046	Deletion of PP_2046 in PP	This study
ΔPP_2047	Deletion of PP_2047 in PP	This study
ΔPP_2048	Deletion of PP_2048 in PP	This study
ΔPP_2049	Deletion of PP_2049 in PP	This study
ΔPP_2051	Deletion of PP_2051 in PP	This study
ΔPP_2047-51	Deletion of PP_2047-51 in PP	This study
ΔPP_3073	Deletion of PP_3073 in PP	This study
Δ*pedS1*	Deletion of *pedS1* in PP	This study
Δ*pedR1*	Deletion of *pedR1* in PP	This study
Δ*pedS2*	Deletion of *pedS2* in PP	This study
Δ*pedR2*	Deletion of *pedR2* in PP	This study
Plasmids		
pQSAK	Suicide plasmid for gene deletion in *Pseudomonas putida* KT2440, Km^R^	(38)
pUCPK/P_*zwf*_*_gfp*	Source for *gfp* gene	(39)
PB1	pUCPK’_P_*pedE*_*_gfp*	This study
PB2	pUCPK’_P_*pedF*_*_gfp*	This study
PB3	pUCPK’_P*pedI_gfp*	This study
PB4	pUCPK’_P_*pedS1*_*_gfp*	This study
PB5	pUCPK’_P_*pedR1*_*_gfp*	This study
PB6	pUCPK’_P_*pedS2/R2*_*_gfp*	This study
PB6_R1	pUCPK’_P_*zwf*_*_pedR1*_P_*pedS2/R2*_*_gfp*	This study
pQE49	pQE80L_P_*T5*__His_PP_2049	This study
PQE73	pQE80L_P_*T5*__His_PP_3073	This study
pU49_1	pUCPK’_P_*lac*__PP_2049	This study
pU49_2	pUCPK’_P_*lac*__UTR1_PP_2049	This study
pU49_3	pUCPK’_P_*lac*_*_*UTR2_PP_2049	This study
pU49_4	pUCPK’_P_*lac*__UTR3_PP_2049	This study
PQ1	pQSAK_*pedE*	This study
PQ2	pQSAK_*pedI*	This study
PQ3	pQSAK_*pedE-I*	This study
PQ4	pQSAK_PP_2046	This study
PQ5	pQSAK_PP_2047	This study
PQ6	pQSAK_PP_2048	This study
PQ7	pQSAK_PP_2049	This study
PQ8	pQSAK_PP_2051	This study
PQ9	pQSAK_PP_3073	This study
PQ10	pQSAK_*pedS1*	This study
PQ11	pQSAK_*pedR1*	This study
PQ12	pQSAK_*pedS2*	This study
PQ13	pQSAK_*pedR2*	This study

### Medium composition and growth conditions

Lysogeny broth (LB) medium was employed to culture the strains. Bacterial cells were cultivated in a modified M9 minimal medium utilizing glucose (or gluconic acid) as the carbon source unless stated otherwise. For degradation experiments, 25 mM of (R)−1,3-BDO, (S)−1,3-BDO, (R,S)−1,3-BDO, or (R)−3-HB was used. The composition of the modified M9 minimal medium is as follows: MgSO_4_·7H_2_O, 0.8 g/L; Na_2_HPO_4_, 18 g/L; (NH_4_)_2_SO_4_, 4.7 g/L; K_2_HPO_4_, 4.05 g/L; and potassium phosphate buffer (pH 7.0), 100 mM. The cells were aerobically cultured in 250 mL flasks containing 20 mL of the working medium, maintained at a temperature of 30°C (or 37°C) with an agitation speed of 200 rpm. The growth of the bacterial cells was monitored by measuring the cell density (cell OD_600_).

### RNA extraction and real-time PCR

The strains were cultured in M9 minimal medium supplemented with 35 mM (R)−1,3-BDO (or (R)−3-HB) or gluconic acid (or glucose) along with (R)−1,3-BDO (or (R)−3-HB). Cultures were incubated aerobically at 30°C and 200 rpm. After 3–6 h of cultivation, approximately 5 5 5 × 10^8^ cells were harvested by centrifugation at 5,000 × *g* for 10 min. Cell pellets were promptly resuspended in 500 μL of RNA solution (Ambion, UK), and RNA extraction was conducted using a total RNA isolation kit (Macherey-Nagel, Germany). For first-strand cDNA synthesis, 1 μg of total RNA was utilized in a 20 µL reaction with the SuperScript III first-strand synthesis system (Invitrogen, USA). Real-time PCR analysis, employing the SYBR green method, was performed in a 20 µL reaction volume using the StepOne Real-Time PCR system (Applied Biosystems, USA). The PCR efficiencies of all primers were experimentally determined for reliable copy-number quantification. mRNA quantity was estimated based on the ΔΔCT value, and assays were conducted in duplicate, with a template-less reaction serving as a negative control.

### GFP fluorescence assay

The recombinant strains, along with different promoter plasmid systems, were introduced into the modified M9 minimal medium, as previously described. Following inoculation, fluorescence and OD_600_ were measured using a Synergy H1 microplate reader (BioTek Instruments, USA). The fluorescence readings were quantified using an excitation wavelength of 486 nm and an emission wavelength of 535 nm. Fluorescence values were normalized to OD_600_ and reported as specific fluorescence (39).

### Transcriptomic analysis

For total RNA isolation, 3 mL of cells from mid-exponential-phase cultures were mixed with 6 mL of RNAprotect Bacteria Reagent (Qiagen). The samples were immediately mixed, incubated at room temperature for 5 min, and centrifuged at 5,000 × *g* for 10 min. The supernatant was decanted, and any residual liquid was removed by inverting the tube onto a paper towel. Total RNA was then extracted using the RNeasy Plus Mini Kit (Qiagen) according to the manufacturer’s instructions. RNA concentration was determined using a NanoDrop 1000 spectrophotometer (Thermo Scientific), and RNA integrity was assessed with the RNA 6000 Pico Kit on an Agilent 2100 Bioanalyzer (Agilent). Ribosomal RNAs were removed using riboPOOLs for pan-prokaryotes (siTOOLs). Following rRNA depletion, paired-end, strand-specific RNA-seq libraries were prepared with the KAPA Stranded RNA-seq Library Preparation Kit (KAPA Biosystems) according to the manufacturer’s instructions. The resulting libraries were analyzed with the Agilent High Sensitivity DNA Kit on an Agilent 2100 Bioanalyzer (Agilent) before sequencing on a HiSeq X platform (Illumina), following the manufacturer’s instructions. All RNA-seq experiments were performed in biological duplicates.

### Enzyme purification and activity measurement

The pQE80L plasmid encoding His-tagged *PP_2049* (or *PP_3073*) was expressed in the wild-type *E. coli* K12 MG1655 strain. Cells were grown at 37°C in LB medium until an optical density of 0.5 (OD_600_) was reached, then induced with 1 mM IPTG. After 6 h of incubation, cells were harvested by centrifugation at 6,000 rpm for 15 min at 4°C. The pellet was washed with phosphate buffer (50 mM, pH 8.0) and disrupted by sonication. The resulting cell-free extract was subjected to purification using Ni-NTA resin according to the manufacturer’s protocol.

HBDH activity was measured following the method of Feller et al. (24) with minor modifications. The reaction was conducted at 37°C and monitored by measuring NADH formation at 340 nm (ε = 6.22 mM⁻¹cm⁻¹). Acetoacetate generation was independently confirmed by HPLC. The assay mixture contained sodium phosphate buffer (50 mM, pH 8.0), substrate (10 mM), and NAD^+^ (0.9 mM). One unit of enzyme activity was defined as the amount of enzyme catalyzing the conversion of 1 µmol of substrate per minute.

### Analytical methods

Cell density was measured at 600 nm with UV/Vis spectrometry (Lambda 20, PerkinElmer, Norwalk, CT, USA). The metabolite concentrations were determined by high-performance liquid chromatography (HPLC; Agilent 1260 Infinity II, USA) as previously described (40).

### Statistical analysis

All data are expressed as the mean ± standard deviation (SD) from three independent biological replicates, unless specified otherwise. Statistical analysis was performed using a paired Student's *t*-test in OriginPro 2020. The following significance thresholds were applied: not significant (ns), *P* ≥ 0.05; *P* < 0.05 (*); *P* < 0.01 (**); and *P* < 0.001 (***).

## Data Availability

The transcriptomic data associated with this study have been deposited in NCBI's Gene Expression Omnibus (41) and are publicly accessible through GEO Series accession number GSE333645.

## SUPPLEMENTAL MATERIAL

Supplemental material aem.00900-26-s0001.pdf Fig. S1 to S10; Table S1.

ASM does not own the copyrights to Supplemental Material that may be linked to, or accessed through, an article. The authors have granted ASM a non-exclusive, world-wide license to publish the Supplemental Material files. Please contact the corresponding author directly for reuse.
