# Supplementary material for: Degradation pathway of (R)-1,3-butanediol in Pseudomonas putida KT2440 and development of its biosensor
Source: Appl Environ Microbiol. 2026 Jun 22;92(7):e00900-26. doi: 10.1128/aem.00900-26 (PMC13390443; doi:10.1128/aem.00900-26)
Supplement: Supplemental material — Fig. S1 to S10; Table S1. [file aem.00900-26-s0001.pdf]

Degradation pathway of (R)-1,3-butanediol in *Pseudomonas putida* KT2440 and development of its biosensor

Nandakumar Arumugam[1], Tayyab Islam[2], Joon Young Park[3], Minchang Jang[1], Mugesh Sankaranarayanan[4], Donghyuk Kim[1], Sung Kuk Lee[1], Sunghoon Park[1, *]

AUTHORS AFFILIATIONS

[1]School of Energy and Chemical Engineering, Ulsan National Institute of Science and Technology, Ulsan 44919, Republic of Korea.

[2]R&D Center, ACTIVON Co., Ltd., Cheongju 28104, Republic of Korea.

[3]Center for Bio-based Chemistry, Korea Research Institute for Chemical Technology, Ulsan 44412, Republic of Korea.

[4]Head – Center for Metabolic Engineering & Synthetic Biology, Department of Biotechnology, Vel Tech Rangarajan Dr. Sagunthala R&D Institute of Science and Technology, Chennai 600062, India.

*Corresponding author: parksh@unist.ac.kr

Supplementary Figures

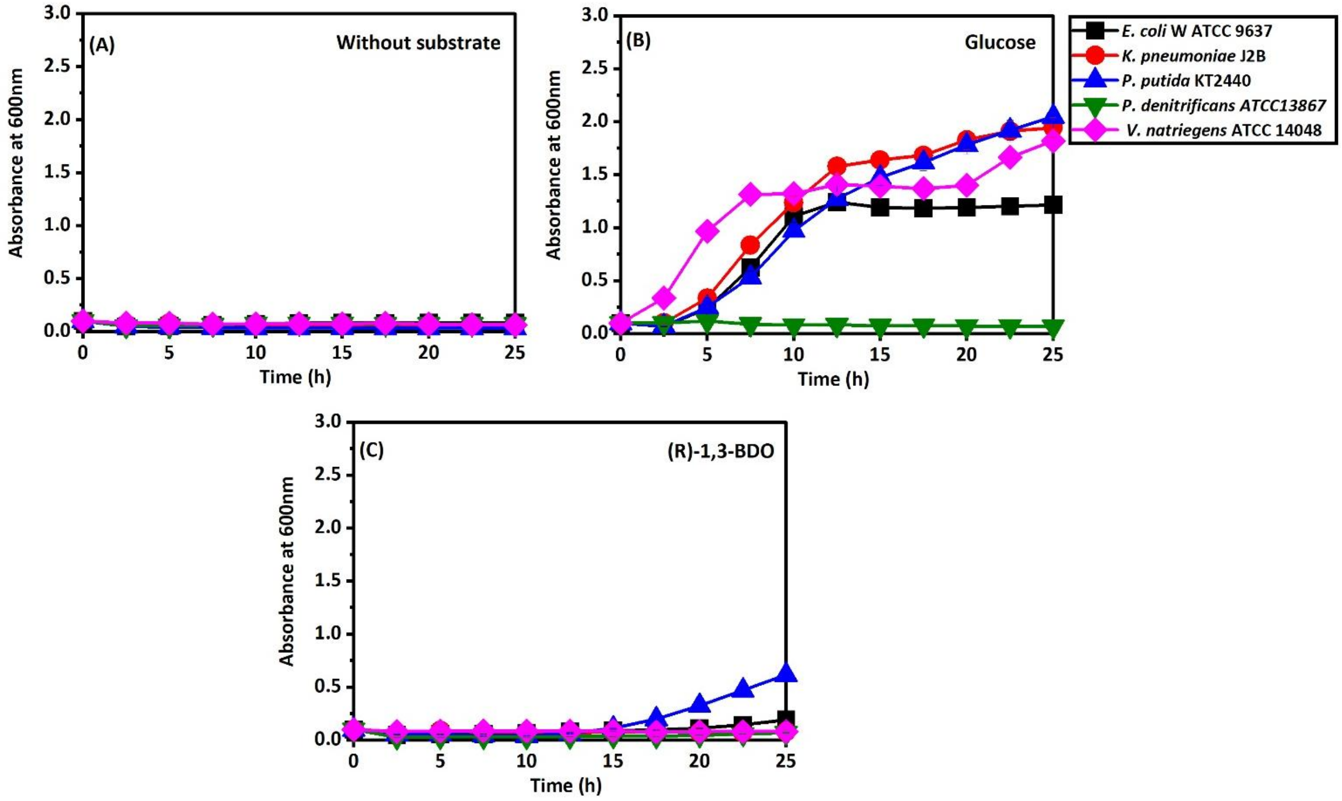

FIG S1 Microbial screening for 1,3-BDO degradation. Growth of selected strains under three conditions: (A) no substrate, (B) 1.5 g/L of glucose, and (C) 25mM (R)-1,3-BDO. Growth was monitored in a 96-well plate using a Synergy H1 microplate reader. Error bars represent SD (n=3).

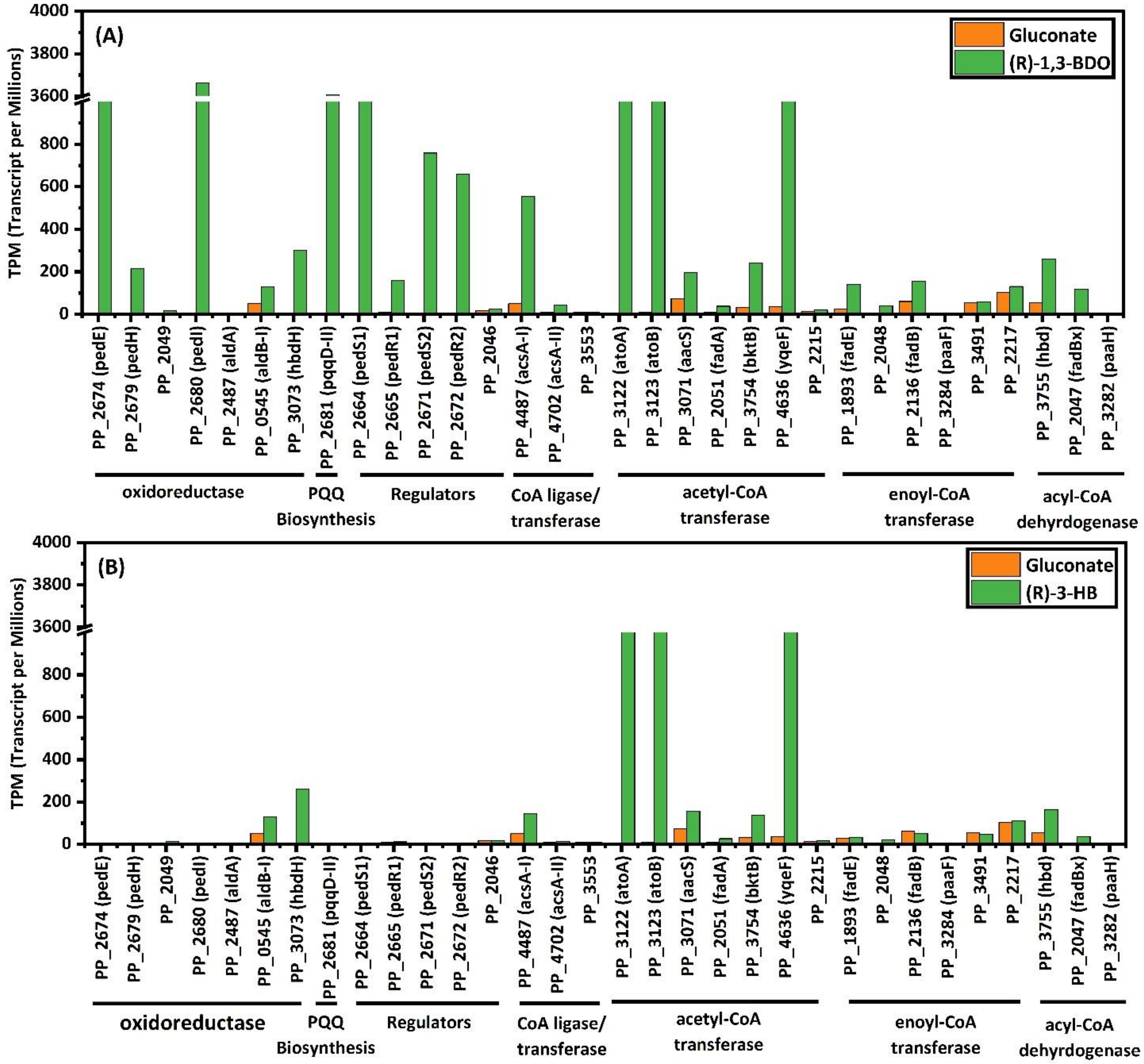

FIG S2 Expression of genes analyzed based on the transcriptomic analysis. (A) (R)-1,3-BDO and (B) (R)-3-HB. Genes are categorized into oxidoreductases, CoA ligases/transferases, acetyl-CoA transferases, eonyl-CoA transferases, and acyl-CoA dehydrogenases.

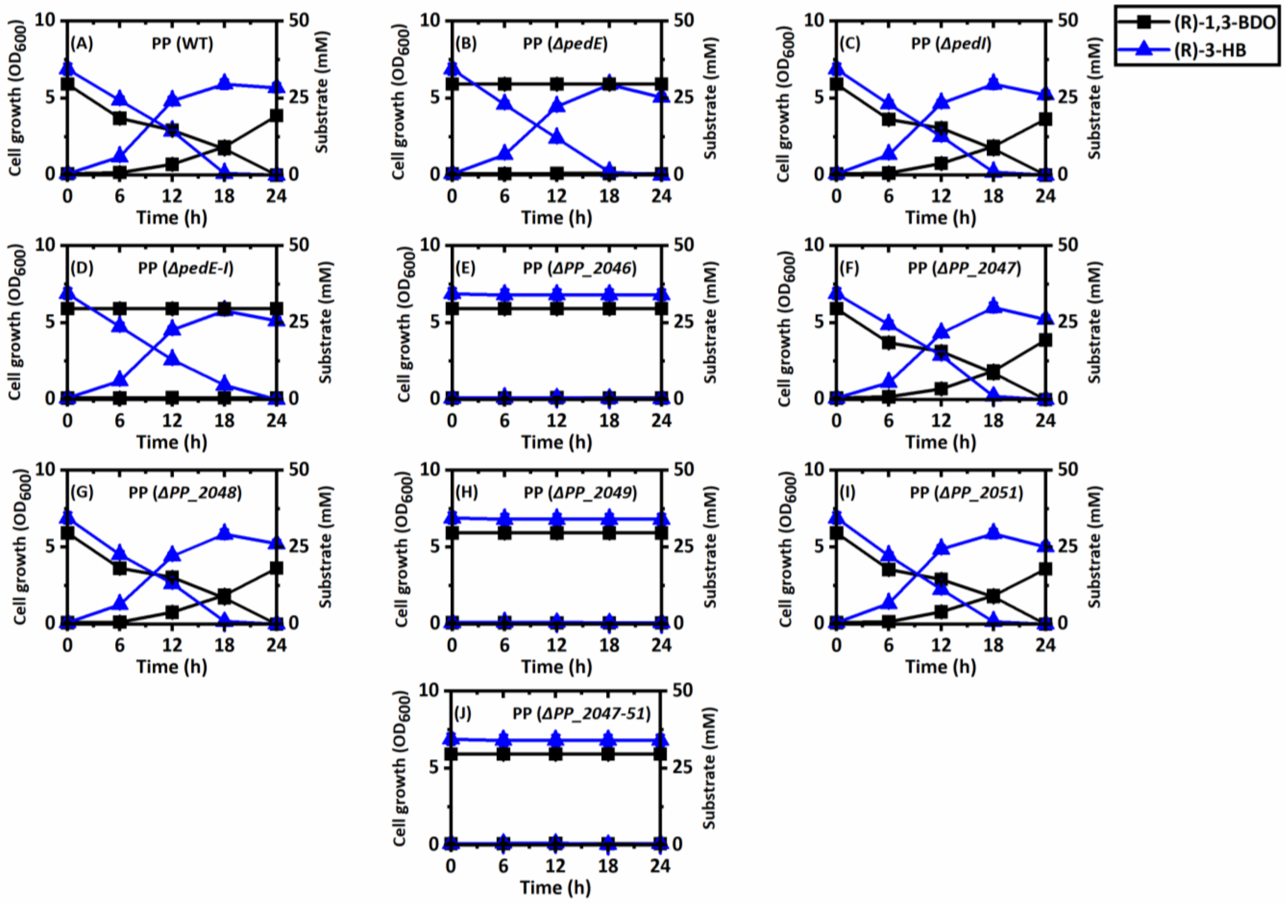

FIG S3 Growth and substrate utilization of deletion mutants. (A-D) Mutants in *ped* cluster genes grow on (R)-1,3-BDO and (R)-3-HB. (E-J) Mutants of additional operons grown on (R)-1,3-BDO and (R)-3-HB. Error bars are presented as mean ± standard deviation (SD) from three biological replicates.

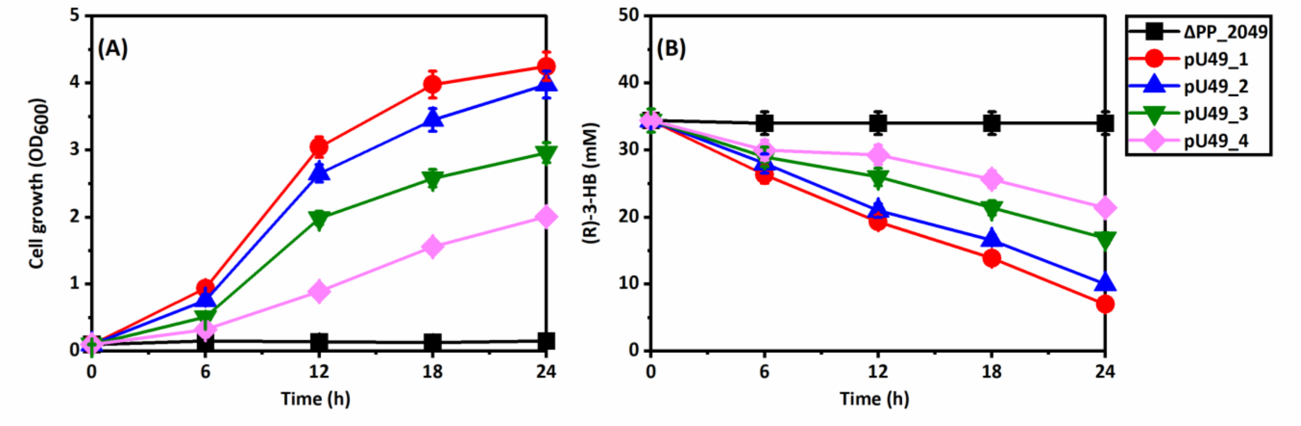

FIG S4 Varied expression of PP_2049 on cell growth (A) and (R)-3-HB consumption (B). Expression of *PP_2049* was modulated in the recombinant strains carrying pUC49 (from pUC49_1 to pUC49_4) by varying UTR strength. Data are presented as mean ± standard deviation (SD) of three independent biological replicates.

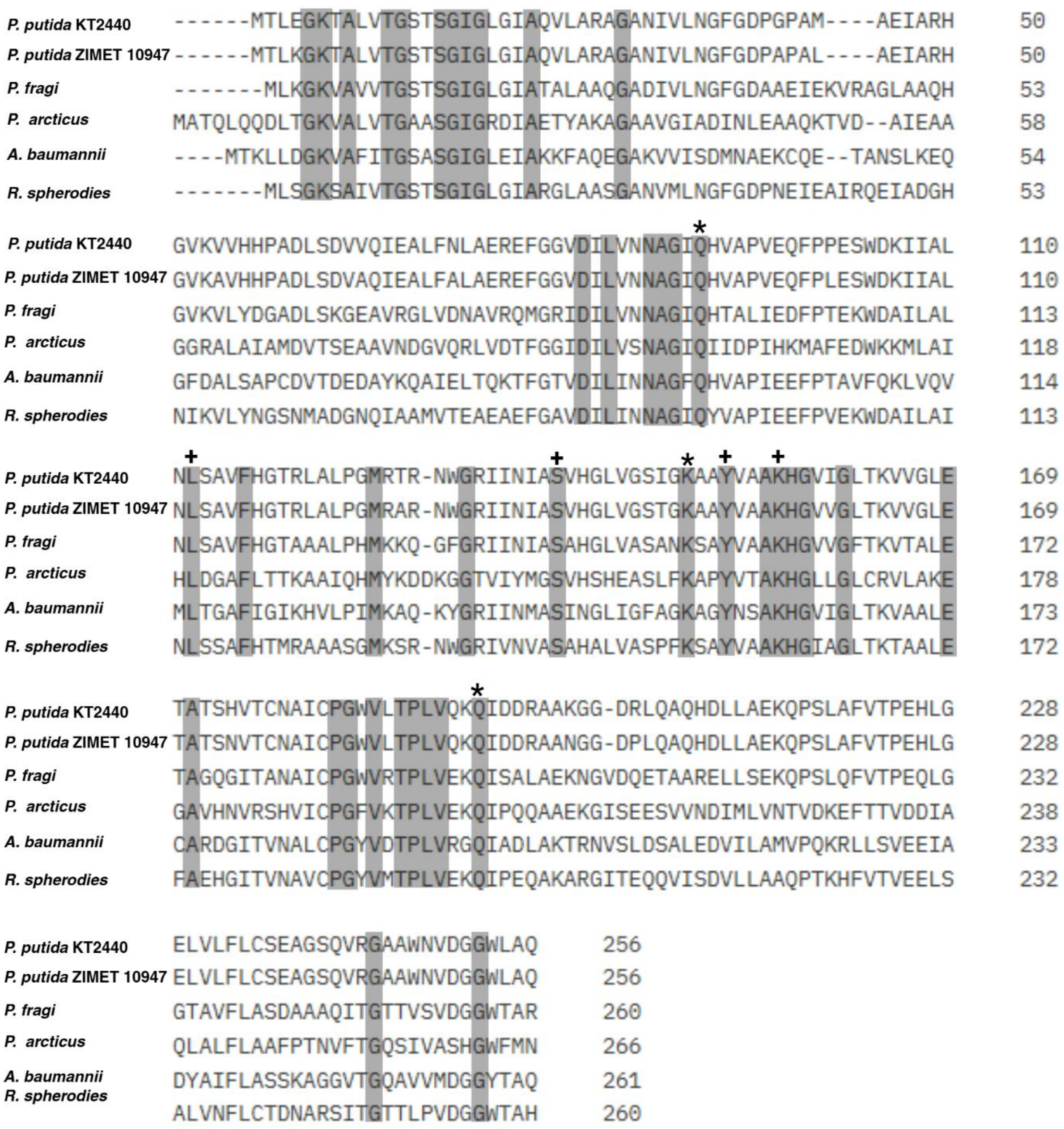

FIG S5 Sequence alignment for bacterial HBDHs from *P. putida* KT2440, *P. putida* ZIMET 10947, *P. fragi, P. arcticus*, *A. baumannii* and *R. spheroides*. Residues shaded in gray are conserved across all strains. The '+' symbol indicates the catalytic tetrad residues; asterisks (∗) denote amino acid positions putatively involved in substrate binding.

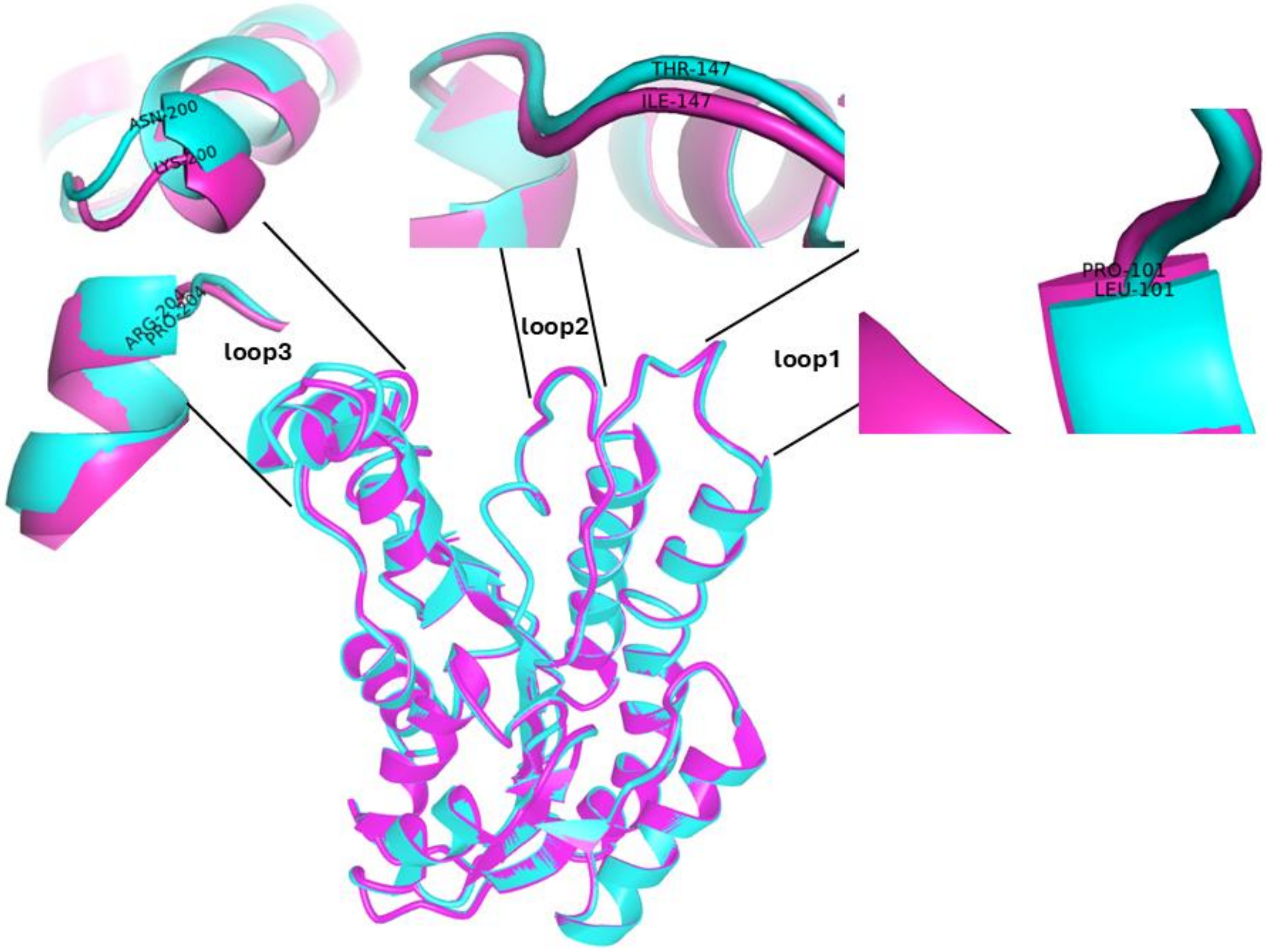

FIG S6 AlphaFold-predicted structural comparison of PP_3073 from *P. putida* KT2440 with BdhA from *P. putida* ZIMET 10947. Superimposition of the predicted protein backbones shows the PP_3073 model (magenta) overlaid on the BdhA structure (cyan). Residues that differ between the two enzymes within the three substrate-binding loop regions are shown as enlarged insets to illustrate the positional and chemical differences at each site. Structures were predicted using AlphaFold2 (1).

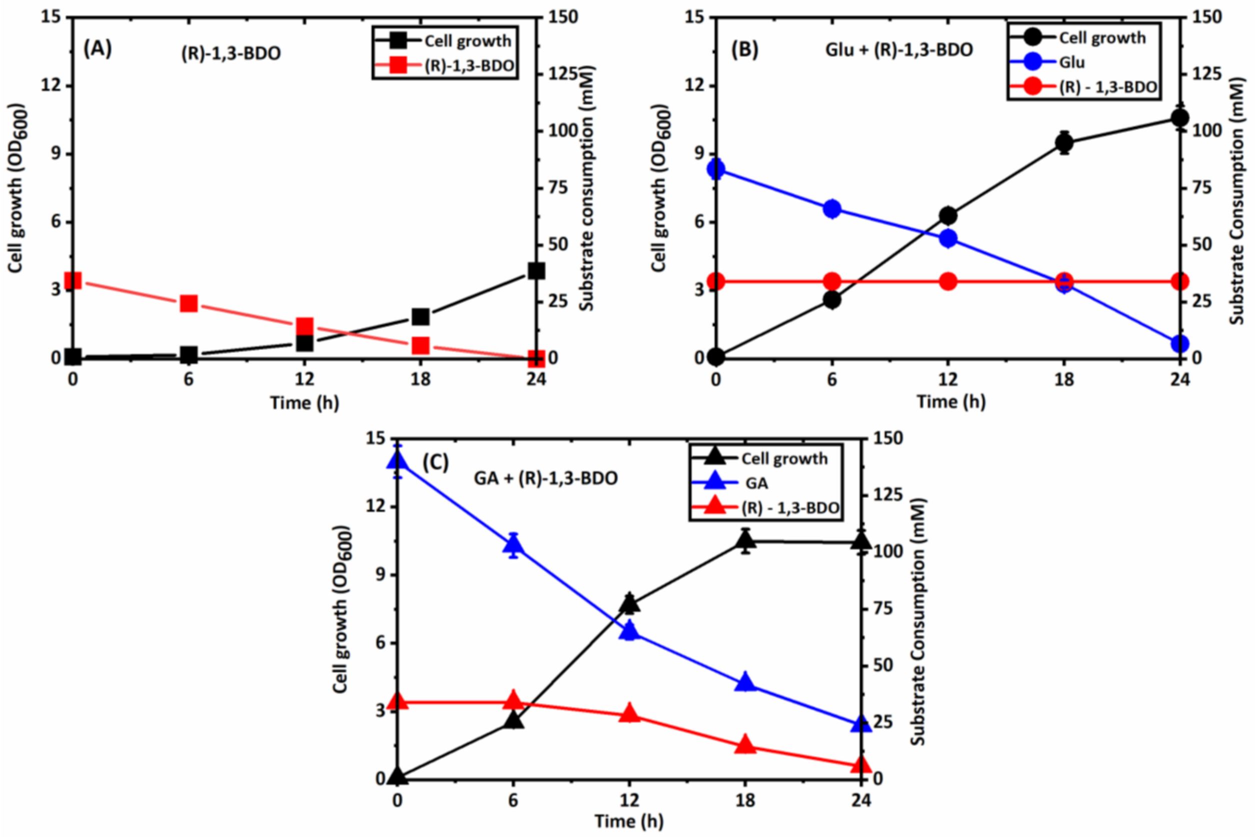

FIG S7 Substrate utilization profiles under different carbon sources. (A) (R)-1,3-BDO only, (B) glucose (glu) + (R)-1,3-BDO, and (C) gluconic acid (GA) + (R)-1,3-BDO. Results from triplicate experiments are shown with SD.

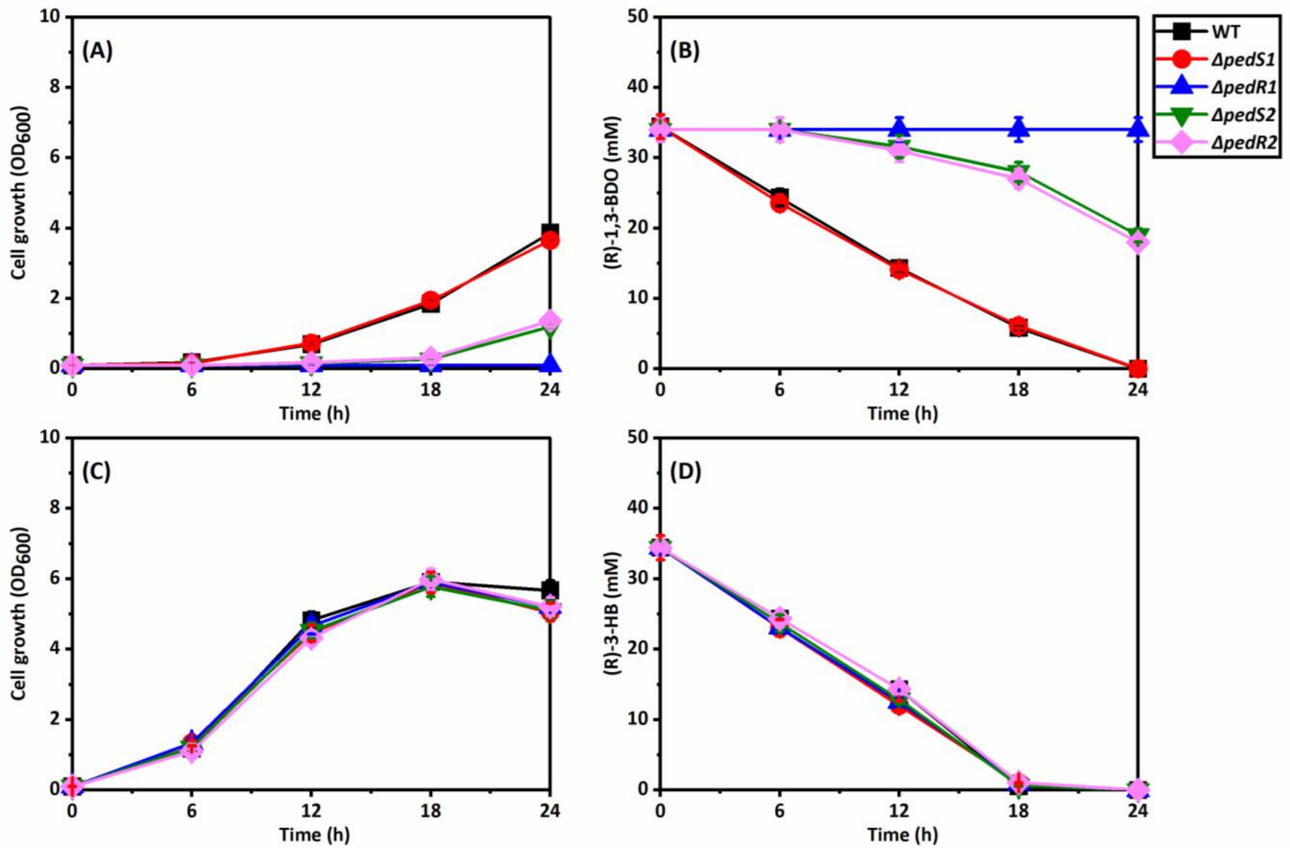

FIG S8 Effect of transcription factor deletions on growth and substrate utilization. (A-B) Cell growth and (R)-1,3-BDO consumption profiles for WT, Δ*pedS1,* Δ*pedR1,* Δ*pedS2,* and Δ*pedR2*. (C-D) Cell growth and (R)-3-HB consumption profiles for the same set of strains. All data shown with SD from triplicate samples.

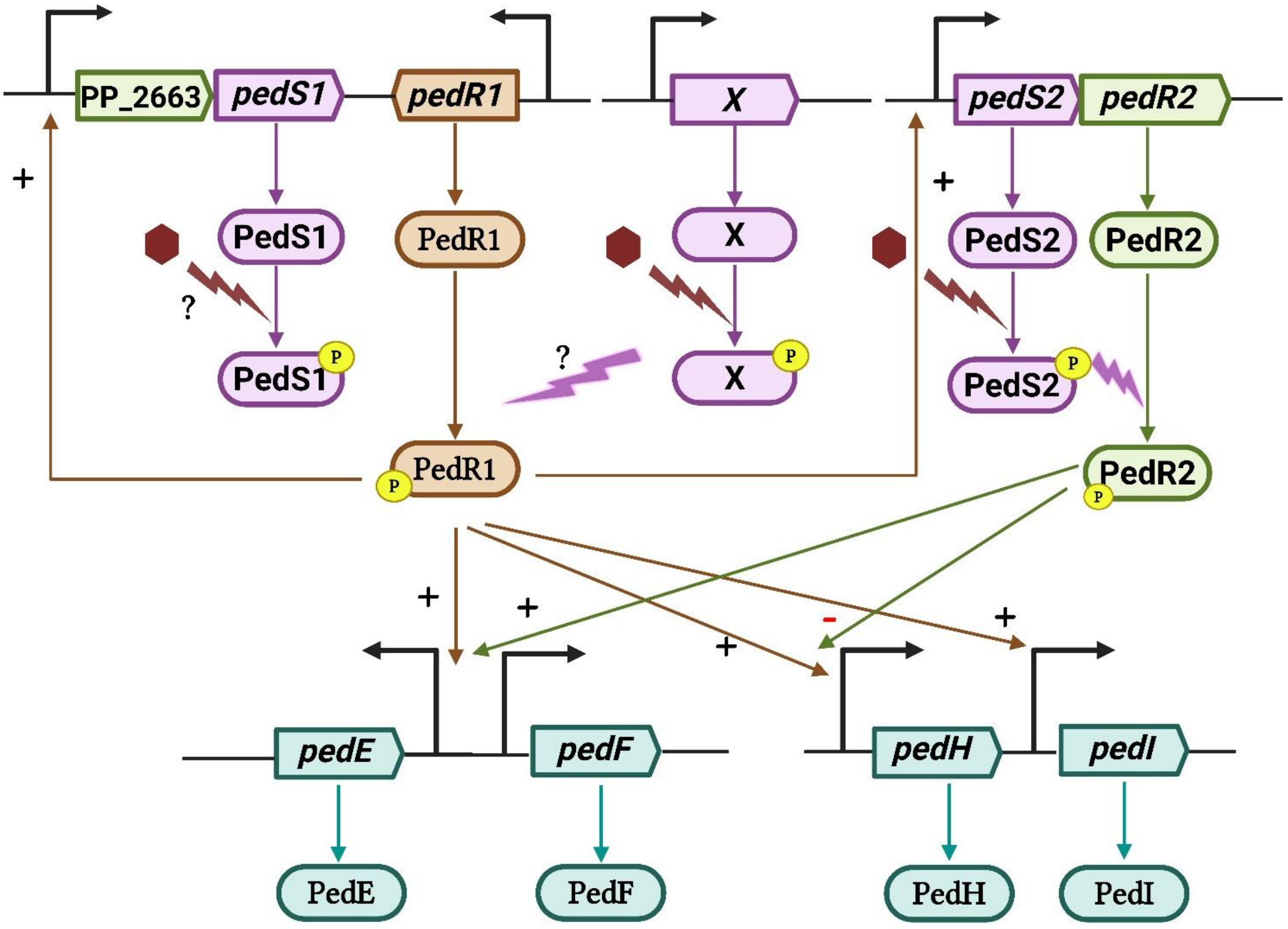

FIG S9 Regulatory mechanisms for (R)-1,3-BDO catabolism in *Pseudomonas putida* KT2440. Expression of *pedR1* appears to be induced by PedS2, or by an unidentified sensor kinase (X). PedR1 activates the expression of *pedS2/R2*. Upon exposure to (R)-1,3-BDO, PedS2 undergoes autophosphorylation and transfers the phosphate group to PedR2. Phosphorylated PedR2, together with PedR1, coordinates the activation of the *ped* catabolic gene clusters needed for (R)-1,3-BDO catabolism. The hexagon symbol represents (R)-1,3-BDO, and the question mark indicates steps in the mechanism that remain unresolved (Modified from (2)).

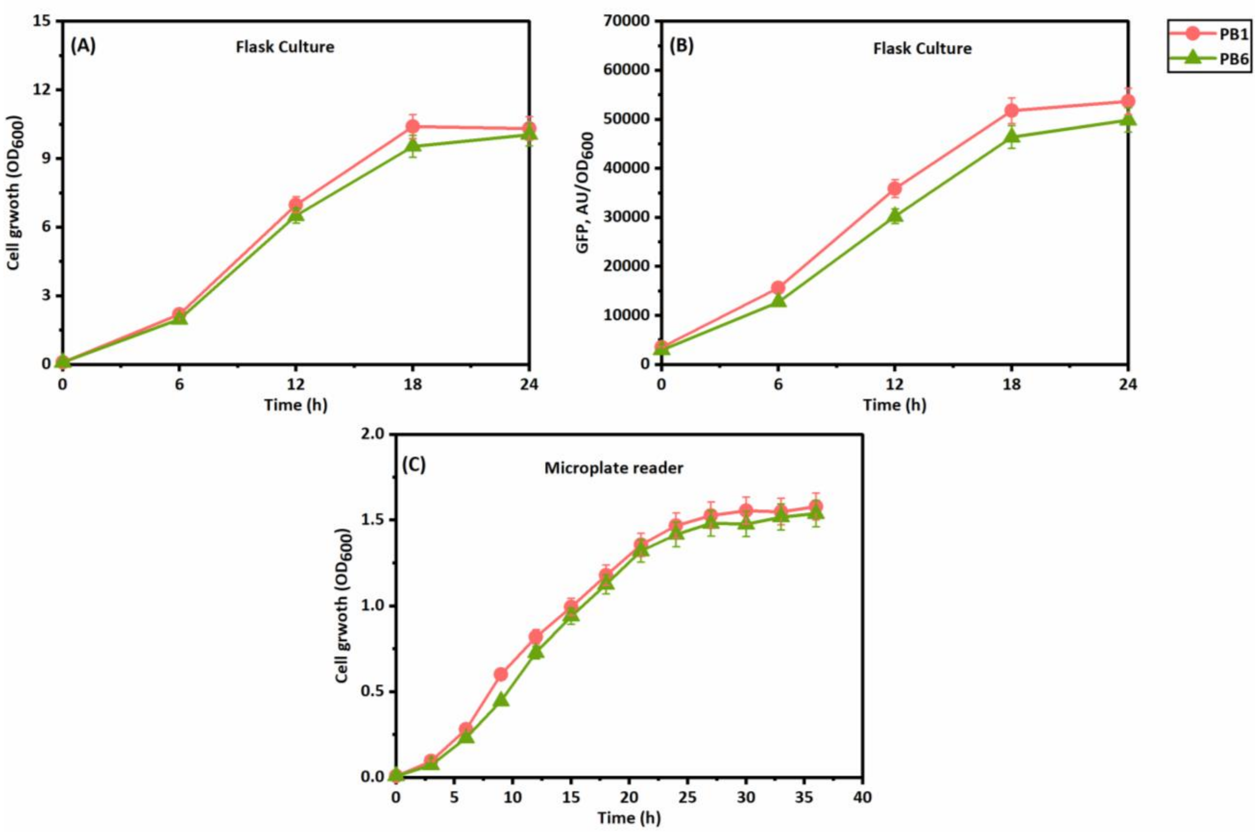

FIG S10 Comparison of flask culture and microplate reader analysis for PB1 and PB6 biosensors. (A) Cell growth, and (B) normalized GFP fluorescence measured in flask cultures. (C) Cell growth was measured using a microplate reader. All experiments are conducted in triplicate, and error bars represent the S.D.

Supplementary Table

Table S1. Kinetic parameters of purified bacterial (R)-3-hydroxybutyrate dehydrogenases (HBDHs)

Source organism	Strain	Size (aa)	k_{cat} (s^{-1})	K_m, substrate (mM)	K_m, cofactor (mM)	Est. sp. act. (U/mg)	Reaction / Substrate	Assay T, pH	Ref.
P. putida	ZIMET 10947	256	1,382	1.5 (3-HB)	0.18 (NAD^+)	~3,117	Oxidation / (R)-3-HB	23°C, pH 8.0	(3)
P. fragi	—	260	285	1.2 (3-HB)	0.13 (NAD^+)	~641	Oxidation / (R)-3-HB	37°C, pH 8.5	(4)
P. putida KT2440	PP_3073	256	—	—	—	0.99	Oxidation / (R)-3-HB	37°C, pH 8.0	This study
P. arcticus	273-4	~260	30 ± 1	0.05 ± 0.01 (AcAc)	0.0064 ± 0.0002 (NADH)	~69[a]	Reduction / AcAc	10°C, pH 7.0	(5-6)
A. baumannii	—	~260	3.4 ± 0.1	0.14 ± 0.01 (AcAc)	0.07 ± 0.01 (NADH)	~7.7[a]	Reduction / AcAc	10°C, pH 7.0	(5-6)
R. spheroides	—	~260[b]	—	0.42 (3-HB)	—	~1.8[c]	Oxidation / (R)-3-HB	25°C, pH 8.5	(7-8)

Footnotes:

[a] Estimated from k_{cat} assuming MW ~27 kDa. Note that PaHBDH and AbHBDH were assayed at 10°C (pH 7.0) for the reduction direction; their k_{cat} values at 25°C would be substantially higher (the Machado et al., 2018 paper shows strong temperature dependence with k_{cat} increasing several-fold between 10°C and 25°C). Thus, direct comparison with the Feller et al., 2006/Ito et al., 2006 oxidation data (23–37°C) should be made with caution.

[b] Pre-genomic era; exact aa count not reported. MW of ~85 kDa for the tetramer by sedimentation equilibrium.

[c] Estimated from the immobilized enzyme activity of 1.2 U/mg (67% of soluble), giving ~1.8 U/mg for the soluble enzyme (8).

REFERENCES

1. Jumper J, Evans R, Pritzel A. *et al.* 2021. Highly accurate protein structure prediction with AlphaFold. Nature 596:583–589. https://doi.org/10.1038/s41586-021-03819-2
2. Promden W, Vangnai AS, Toyama H, Matsushita K, Pongsawasdi P. 2009. Analysis of the promoter activities of the genes encoding three quinoprotein alcohol dehydrogenases in *Pseudomonas putida* HK5. Microbiol 155:594–603. https://doi.org/10.1099/mic.0.021956-0
3. Feller C, Günther R, Hofmann H, Grunow M. 2006. Molecular Basis of Substrate Recognition in D-3-hydroxybutyrate dehydrogenase from *Pseudomonas putida*. ChemBioChem 7:1410–1418. https://doi.org/10.1002/cbic.200600167
4. Ito K, Nakajima Y, Ichihara E, Ogawa K, Katayama N, Nakashima K, Yoshimoto T. 2006. D-3-hydroxybutyrate dehydrogenase from *Pseudomonas fragi*: Molecular cloning of the enzyme gene and crystal structure of the enzyme. J Mol Biol 355:722–733. https://doi.org/10.1016/j.jmb.2005.10.072
5. Machado TFG, Purg M, McMahon SA, Read BJ, Oehler V, Åqvist J, Gloster TM, da Silva RG. 2020. Dissecting the Mechanism of (*R*)-3-Hydroxybutyrate Dehydrogenase by Kinetic Isotope Effects, Protein Crystallography, and Computational Chemistry. ACS Catal. 10(24):15019-15032. https://doi.org/10.1021/acscatal.0c04736
6. Machado TFG, Gloster TM, da Silva RG. 2018. Linear Eyring Plots Conceal a Change in the Rate-Limiting Step in an Enzyme Reaction. Biochem. 57(49):6757-6761. https://doi.org/10.1021/acs.biochem.8b01099
7. Bergmeyer HU, Gawehn K, Klotzsch H, Krebs HA, Williamson DH. 1967. Purification and properties of crystalline 3-hydroxybutyrate dehydrogenase from *Rhodopseudomonas spheroides*. Biochem J 102(2):423-431. https://doi.org/10.1042/bj1020423
8. Preuveneers MJ, Peacock D, Crook EM, Clark JB, Brocklehurst K. 1973. d-3-Hydroxybutyrate dehydrogenase from *Rhodopseudomonas spheroides*. Kinetic mechanism

from steady-state kinetics of the reaction catalysed by the enzyme in solution and covalently attached to diethylaminoethylcellulose. Biochem J 133: 133-157. https://doi.org/10.1042/bj1330133
